# Optimized p53 immunohistochemistry is an accurate predictor of *TP53* mutation in ovarian carcinoma

**DOI:** 10.1002/cjp2.53

**Published:** 2016-07-13

**Authors:** Martin Köbel, Anna M Piskorz, Sandra Lee, Shuhong Lui, Cecile LePage, Francesco Marass, Nitzan Rosenfeld, Anne‐Marie Mes Masson, James D Brenton

**Affiliations:** ^1^ Department of Pathology and Laboratory Medicine University of Calgary Calgary AB Canada; ^2^ Cancer Research UK Cambridge Institute, University of Cambridge, Li Ka Shing Centre Cambridge CB2 0RE UK; ^3^ Centre de recherche du Centre hospitalier de l'Université de Montréal (CRCHUM) Montreal QC Canada; ^4^ Institut du cancer de Montréal, Montreal QC Canada

**Keywords:** p53, *TP53*, high grade serous ovarian carcinoma, endometrioid ovarian carcinoma, immunohistochemistry, prediction, biomarker, next‐generation‐sequencing

## Abstract

*TP53* mutations are ubiquitous in high‐grade serous ovarian carcinomas (HGSOC), and the presence of *TP53* mutation discriminates between high and low‐grade serous carcinomas and is now an important biomarker for clinical trials targeting mutant p53. p53 immunohistochemistry (IHC) is widely used as a surrogate for *TP53* mutation but its accuracy has not been established. The objective of this study was to test whether improved methods for p53 IHC could reliably predict *TP53* mutations independently identified by next generation sequencing (NGS). Four clinical p53 IHC assays and tagged‐amplicon NGS for *TP53* were performed on 171 HGSOC and 80 endometrioid carcinomas (EC). p53 expression was scored as overexpression (OE), complete absence (CA), cytoplasmic (CY) or wild type (WT). p53 IHC was evaluated as a binary classifier where any abnormal staining predicted deleterious *TP53* mutation and as a ternary classifier where OE, CA or WT staining predicted gain‐of‐function (GOF or nonsynonymous), loss‐of‐function (LOF including stopgain, indel, splicing) or no detectable *TP53* mutations (NDM), respectively. Deleterious *TP53* mutations were detected in 169/171 (99%) HGSOC and 7/80 (8.8%) EC. The overall accuracy for the best performing IHC assay for binary and ternary prediction was 0.94 and 0.91 respectively, which improved to 0.97 (sensitivity 0.96, specificity 1.00) and 0.95 after secondary analysis of discordant cases. The sensitivity for predicting LOF mutations was lower at 0.76 because p53 IHC detected mutant p53 protein in 13 HGSOC with LOF mutations. CY staining associated with LOF was seen in 4 (2.3%) of HGSOC. Optimized p53 IHC can approach 100% specificity for the presence of *TP53* mutation and its high negative predictive value is clinically useful as it can exclude the possibility of a low‐grade serous tumour. 4.1% of HGSOC cases have detectable WT staining while harboring a *TP53* LOF mutation, which limits sensitivity for binary prediction of mutation to 96%.

## Introduction

High‐grade serous ovarian carcinoma (HGSOC) is the most aggressive histological type of ovarian carcinoma and pathogenic *TP53* mutation is present in >96% of cases 1. *TP53* mutation is frequently present in fallopian tube precursor lesions suggesting that it is an early driver event 2, 3, 4. HGSOC show remarkable intratumoural genetic heterogeneity characterized by divergent copy number abnormalities and frequent passenger substitutions – however owing to their presence in the ancestral clone, *TP53* mutations are detectable in all subclones of an individual's HGSOC 5, 6, 7. The ubiquitous presence of *TP53* mutations in HGSOC provides an important diagnostic feature for small tissue biopsies, particularly in distinguishing low‐grade serous carcinoma from HGSOC, and has been used for personalized disease monitoring using circulating tumour DNA in plasma samples 8, 9, 10, 11.

Despite the clinical importance of *TP53* mutation, rapid sequencing is not widely available and immunohistochemistry (IHC) remains the commonest method to infer *TP53* mutational status. However, the accuracy of IHC as a predictor of *TP53* mutation in ovarian carcinoma has not been precisely defined. Early studies showed only a modest correlation between p53 staining pattern and *TP53* mutation although these results were based on limited somatic sequencing or only scored overexpression of p53 12, 13, 14, 15, 16, 17. We have previously proposed a 3‐tier scoring system to describe p53 staining in ovarian carcinoma: overexpression (OE), complete absence (CA) or wild‐type (WT) 18. OE is most commonly associated with nonsynonymous *TP53* mutations, which interfere with MDM2‐induced ubiquitination and degradation of p53, resulting in excessive p53 protein accumulation in the nucleus. CA is associated with nonsense mutations, which introduce a premature stop codon that triggers nonsense‐mediated RNA decay, or indel and splice acceptor mutations that interfere with correct protein translation by introducing frame shifts or aberrant splicing. WT expression is characterized by a variable staining intensity in a variable number of tumour cell nuclei. Depending on the proliferation and maturation status of tumour cells, the number of variably intense staining nuclei can range from a few to even the majority. Interpretation with the 3‐tier pattern increases the sensitivity of IHC and abnormal p53 staining was observed in 88–94% of HGSOC as compared to 14% of endometrioid ovarian carcinoma (EC) 18, 19. However, comparison of the 3‐tier pattern to *TP53* mutation in a study of 57 ovarian carcinomas showed that abnormal p53 expression predicted pathogenic mutation with a sensitivity of 94% but a specificity of only 38% 20.

The functional consequences of *TP53* mutations have been divided into two classes with distinct biological effects (reviewed in 21). Nonsynonymous mutations have been shown to induce gain of function (GOF) class effects including metabolic reprogramming, chromatin reorganization and increased motility and invasion 22, 23. Loss of function (LOF) class mutations, that include stopgain, frameshift and splicing mutations, have weaker tumourigenic effects than GOF mutations in genetically engineered mouse models 24, 25. Distinguishing between GOF and LOF mutations may be clinically important in HGSOC as LOF mutations have been associated with reduced overall survival 18, 26, 27. In addition, the emergence of new clinical trials testing strategies to restore wild‐type p53 conformation 28, 29, 30, 31, or to reinstate protein translation of LOF mutations 32, 33 emphasizes the need for robust and reliable IHC biomarkers for p53.

The primary aim of this study was to determine the sensitivity and specificity of IHC to predict the presence and class of *TP53* mutation. We compared clinically relevant assays for p53 staining to next generation sequencing of tumour tissue as the gold standard reference. The secondary aim was to investigate misclassified cases to categorize *TP53* mutations with unexpected patterns of p53 staining.

## Methods

### Study cohort and DNA extraction

This study was granted ethical approval REB15‐0945. The study cohort was sourced from the Canadian Ovarian Experimental Unified Resource (COEUR) 19, 34 and the Pathology Department of Calgary Laboratory Services (CLS) 35. All cases underwent additional staining with histotype‐specific IHC as part of detailed pathological review to assign the correct ovarian histotype 19. Initial sequencing analysis was carried out on fresh‐frozen tumour tissue from the COEUR cohort and formalin‐fixed paraffin embedded (FFPE) tissue from the CLS cohort. DNA was extracted from fresh‐frozen material as previously described 34. For FFPE material, DNA was extracted from two 1 mm tissue microarray cores using the QIAam Micro DNA kit (Qiagen) following the manufacturer's protocol except that additional incubation with lysis buffer was performed at 95°C for 15 min before adding proteinase K.

### Tagged‐amplicon sequencing

*TP53* was sequenced starting with amplification of the entire coding sequence exon 2–11 of *TP53* with flanking splice sites using tagged‐amplicon sequencing with the Fluidigm Access Array 48.48 platform as described previously 9. Tagged‐amplicon sequencing libraries were sequenced on the Illumina HiSeq2000 or MiSeq platforms using paired‐end 100bp reads (primer sequences available upon request). Sequencing data and variant verification were performed using an in‐house analysis pipeline and IGV software as described 9, 36.

### Sanger sequencing

The *TP53* coding sequences (exons 2–11) were amplified as described 37 with the following modifications: PCR reactions were performed in 25 μl, universal primers M13 forward and M13 reverse were incorporated into primer pairs and used to sequence in both the forward and reverse directions. To sequence exon 7, an alternative forward primer was used (*TP53*‐7F: CAGGTCTCCCCAAGGCGC AC) to avoid a poly A tract downstream of the exon 7 forward primer described in 37 (CATCCTGGCT AACGGTGAAAC). Mutational analysis was performed using Mutation Surveyor Software version 4.0.4 (SoftGenetics) using default settings.

### Immunohistochemistry

Tissue microarrays were constructed as described 34 using 0.6 mm cores from FFPE archival tumour tissue. Pathological scoring was performed by sub‐specialized gynaecological pathologists (MK, SL). Four methods for clinical immunohistochemical analysis of p53 staining were performed as described 18, 19, 34, 38 using 4 µm sections from tissue microarrays and tumour blocks.

### Statistical analysis

The diagnostic test performance of p53 IHC was quantified by calculating sensitivity, specificity and accuracy using *TP53* mutation status as the reference. Two p53 IHC classifications were evaluated: (1) binary classification where abnormal or normal staining was compared to the presence or absence of a deleterious *TP53* mutation and (2) ternary classification where OE, CA or WT staining was compared to GOF, LOF mutation classes or cases with no detectable mutation (NDM), respectively (GOF for any nonsynonymous mutation, LOF for any stopgain, indel or splicing mutation and NDM for normal or synonymous mutations). Cases that were not assessable for IHC were excluded from comparison. Cases with cytoplasmic staining were excluded from ternary classification. Statistical analyses were performed using the R statistical language 39 and classification analysis was performed using the caret package 40. The complete statistical analysis is provided in the Supplementary Analytical File as a knitr document which fully reproduces the analyses 41.

## Results

**Figure 1:**
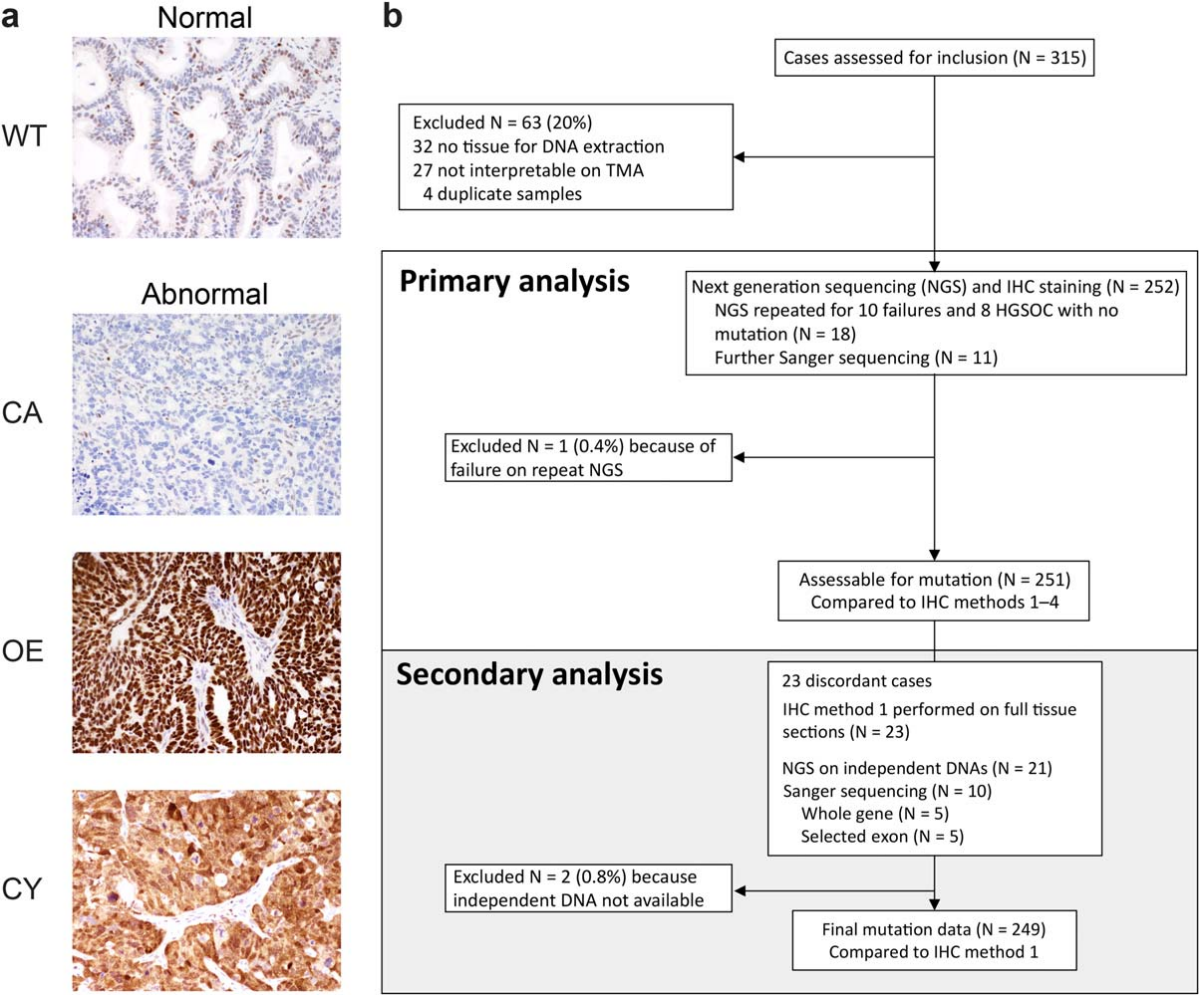
p53 scoring system and flow of samples through study. (a) p53 IHC scoring system: Normal or wild type pattern (WT) is characterized by variable staining intensity. Abnormal overexpression (OE) shows strongly intense staining in all tumour cell nuclei. Abnormal complete absence (CA) shows complete absence of expression within tumour cell nuclei. Note the variable intensity of normal p53 expression seen in fibroblasts and lymphocytes which act as an intrinsic control. Abnormal cytoplasmic staining (CY) shows diffuse cytoplasmic staining in the absence of strong nuclear staining. (b) Flow of samples through the study.

The study design selected HGSOC and EC cases for *TP53* sequencing and p53 IHC from two ovarian carcinoma cohorts available on tissue microarrays that had been subjected to detailed pathology review and immunophenotyping to accurately determine histotype. For the primary analysis, IHC and sequencing results were independently generated and the interpretation of mutation and staining pattern was blinded. Figure 1a shows the staining patterns recognized for p53 IHC scoring.

### *TP53* mutation analysis

Figure 1b shows the flow of samples through the study. 315 cases of HGSOC and EC represented on three tissue microarrays were initially selected for eligibility, from which 252 (80%) DNAs were subjected to tagged‐amplicon sequencing. Supplementary material, Figure S1 shows the sequencing strategy for mutation detection. After quality control (supplementary material, Table S1), 251 cases were evaluable for mutation status (99.6%), which consisted of 171 HGSOC and 80 EC cases. Median sequencing depth for *TP53* was estimated as ×3690 (IQR 1756–6913) and median *TP53* mutant allelic fraction was 0.65 (IQR 0.45–0.78).

**Table 1:** Study demographics

	HGSOC	EC
*N* (total = 251)	171	80
Age (median, IQR)	58 (52–67)	55 (49–66)
Stage		
I	7 (4%)	50 (63%)
II	7 (4%)	19 (24%)
III	113 (66%)	6 (8%)
IV	31 (18%)	2 (3%)
NA	13 (8%)	3 (4%)

HGSOC, high‐grade serous ovarian carcinoma; EC, endometrioid carcinoma; IQR, interquartile range; NA, not available.

**Figure 2:**
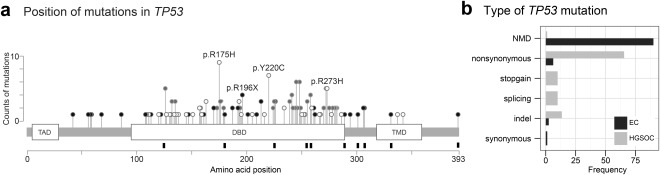
Frequency and position of *TP53* mutations. (a) Schematic of the *TP53* gene showing protein domains (open boxes) with lollipops showing positions and counts of identified mutations. Mutation type is indicated by circle fill: white, non‐synonymous; black, indel or in‐frame; grey, codons with >1 mutation type. Black rectangles below the cartoon show codon positions of *TP53* mutations with discordant p53 IHC results. (TAD, transactivation domain; DBD, DNA binding domain; TMD, tetramerization domain). (b) Barplot showing the frequency and type of *TP53* mutation by histotype.

Table 1 shows the clinical features for the evaluable 251 cases. *TP53* mutations were detected in 177 (71%) cases, which included one synonymous mutation in an EC that was not considered deleterious. The most common amino acid substitutions were p.R175H (*N* = 9), p.Y220C (*N* = 6), p.R273H (*N* = 5) and p.R196X (*N* = 4) (Figure 2a). Deleterious *TP53* mutations were present in 169 (99%) HGSOC and 7 (8.8%) EC cases. In HGSOC cases there were 112 (66%) GOF and 57 (33%) predicted LOF mutations. The EC cases had 5 (6.3%) GOF and 2 (2.5%) predicted LOF mutations (Figure 2b).

### Immunohistochemical staining for p53

**Figure 3:**
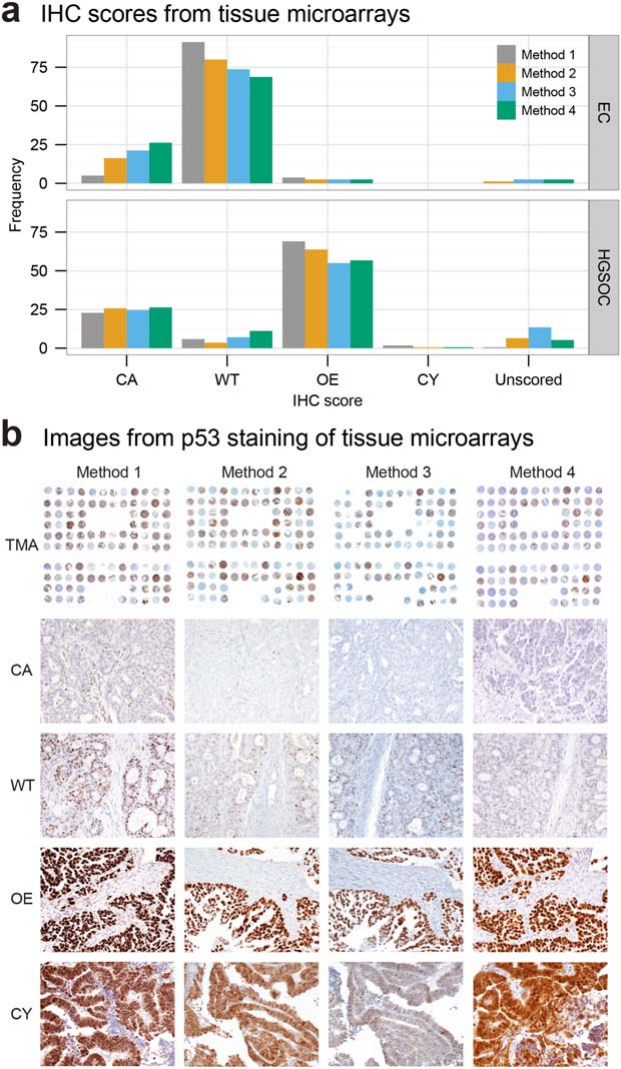
Tissue microarray analysis of p53 IHC staining. (a) Barplot showing the frequency of p53 IHC staining patterns in endometrioid (EC; *N* = 80) and high‐grade serous ovarian carcinoma (HGSOC; *N* = 171) cases. Staining is scored as follows: complete absence (CA) of expression in tumour cells; wild‐type (WT) pattern **showing** nuclear staining with variable intensity in 1–80% of tumour cell nuclei; overexpression (OE) showing nuclear staining with strong intensity in >80% of tumour cell nuclei; strong cytoplasmic (CY) staining with absent nuclear staining; unscored for non‐assessable cores. (b) Representative tissue microarray images showing p53 immunohistochemical staining patterns.

Four IHC assays commonly used for clinical reporting of p53 status were selected for comparison (Table 2). Inspection of the 3‐tier scores for p53 staining across 171 HGSOC and 80 EC cases on TMAs revealed systematic differences in the performance of the four assays (Figure 3a and Supplementary Analytical File). Three cases showed strong cytoplasmic staining (CY) without nuclear overexpression and were scored separately (discussed below). Inspection of the IHC results for EC cases, where a low frequency of *TP53* mutation was expected, showed that scoring for WT was highest for method 1 (91%, N=73) and was progressively lower with methods 2–4 (Figure 3a). For scoring CA, method 1 had the lowest frequency in EC (5%, *N* = 4). For the HGSOC cases, where ubiquitous mutation was expected, method 1 had the highest frequency for scoring of OE (69%, *N* = 118) and the lowest for CA (23%, *N* = 39). Cross‐comparison of the TMA images suggested that method 1 had the strongest staining for p53 across EC and HGSOC cases (Figure 3b) and that scoring of the weaker staining from methods 2–4 frequently shifted interpretation of cases from WT to CA (supplementary material, Figure S2 and Supplementary Analytical File). Inter‐rater variability for scoring with method 1 by a second, independent observer showed very good agreement ( *κ* = 0.88; Cohen's Kappa for two raters with equal weights, *N* = 148, *p* ≪ 0.001). The contingency table for the two observers’ scores showed that disagreement most commonly occurred for CA and WT staining (Supplementary Analytical File).

**Table 2:** Immunohistochemical assays

IHC method	Antibody (Supplier)	IHC Platform	Dilution	Pretreatment
1	DO‐7 (DAKO)	Leica Bond Max	1:2500	ER2
2	DO‐1 (Santa Cruz)	Ventana Discovery Ultra	1:200	CC2
3	DO‐7 (DAKO)	Ventana Discovery Ultra	1:400	CC1
4	E26 (Epitomics)	DAKO Plus Autostainer	1:100	pH6

### Primary analysis of p53 IHC as predictor of *TP53* mutation class

**Figure 4:**
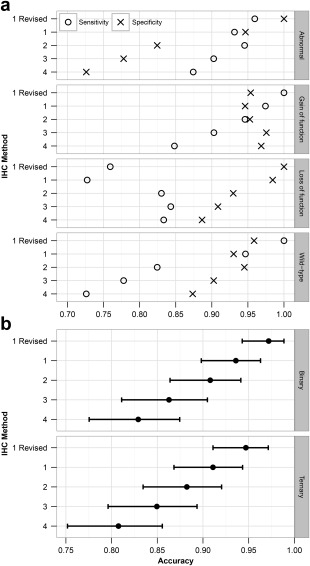
Analysis of IHC method to predict presence of *TP53* mutation. (a) Cleveland dot plot showing the sensitivity and specificity for binary and ternary predictions of the class of *TP53* mutations. (b) Dot plot showing the overall accuracy of IHC methods for binary and ternary predictions. Error bars show 95% confidence limits.

The performance of p53 IHC in predicting *TP53* mutation was tested both as (1) a binary classifier for any pathogenic *TP53* mutation and (2) as a ternary classifier where OE, CA or WT staining was compared to GOF, LOF *TP53* mutations or NDM. The sensitivity and specificity results for these comparisons are shown in Figure 4 and Table 3 (see also Supplementary Analytical File). For binary classification, IHC method 2 and 1 had the highest sensitivity and there was a progressive increase in specificity from method 4 to method 1. For ternary classification, method 1 had highest sensitivity for GOF mutations and was markedly better for WT predictions (Figure 4a). However, the sensitivity for method 1 for prediction of LOF mutation was markedly reduced compared to other methods. For both binary and ternary classification, method 1 had the highest overall accuracy (Figure 4b).

**Table 3:** Concordance of p53 IHC with *TP53* mutation

Mutation type	IHC method	Sensitivity	Specificity	Balanced accuracy
Binary	1	0.93	0.95	0.94
	2	0.95	0.82	0.89
	3	0.90	0.78	0.84
	4	0.87	0.73	0.80
	1 Revised	0.96	1.00	0.98
Gain of function	1	0.97	0.95	0.96
	2	0.95	0.95	0.95
	3	0.90	0.98	0.94
	4	0.85	0.97	0.91
	1 Revised	1.00	0.95	0.98
Loss of function	1	0.73	0.98	0.86
	2	0.83	0.93	0.88
	3	0.84	0.91	0.88
	4	0.83	0.89	0.86
	1 Revised	0.76	1.00	0.88
NDM	1	0.95	0.93	0.94
	2	0.82	0.95	0.88
	3	0.78	0.90	0.84
	4	0.73	0.87	0.80
	1 Revised	1.00	0.96	0.98

NDM, no detectable mutation.

### Secondary analysis

Twenty‐three (9%) cases were discordant between the class of *TP53* mutation and the IHC staining pattern (supplementary material, Table S2). These cases were subjected to an independent second analysis with repeat sequencing from new DNA samples and IHC staining on whole sections from the original tissue blocks using method 1. From 21 evaluable sequences, the mutation result was revised in two cases from nonsynonymous to NDM which was in agreement with the IHC staining. The IHC staining was revised in five cases from staining whole sections: WT to CA (*N* = 1), WT to OE (*N* = 1) and CA to WT (*N* = 3) (supplementary material, Figure S3).

The revised data for method 1 was then used to re‐estimate the classifier performance for method 1. The accuracy of the binary classifier to predict any *TP53* mutation increased from 0.94 to 0.97 (Figure 4b) with sensitivity of 0.96 and specificity of 1.00. The accuracy to predict GOF and NDM increased (Table 3) but the sensitivity to detect LOF mutations remained low (0.76).

### Discordant cases

Comparison of the data from primary and secondary analysis showed that 13 HGSOC cases that were predicted to have LOF mutations did not show the expected CA pattern (Table 4). The observed staining patterns (OE, *N* = 6; WT, *N* = 7) were consistent across multiple experiments, suggesting that these results did not simply arise from mistakes in interpretation.

**Table 4:** Concordance of p53 expression from IHC method 1 with *TP53* mutation status in all cases (HGSOC+EC) after secondary analysis

IHC	Nonsynonymous	Indel	Stopgain	Splicing	NDM	Total
OE	115	2	2	2	0	121
CA	0	16	13	12	0	41
CY	0	2	2	0	0	4
WT	0	4	0	3	76	83
Total	115	24	17	17	76	249

OE, p53 overexpression; CA, p53 complete absence of expression; WT, p53 wild type pattern of expression; NDM, no detectable mutation; HGSOC, high‐grade serous ovarian carcinoma; EC, endometrioid carcinoma.

**Figure 5:**
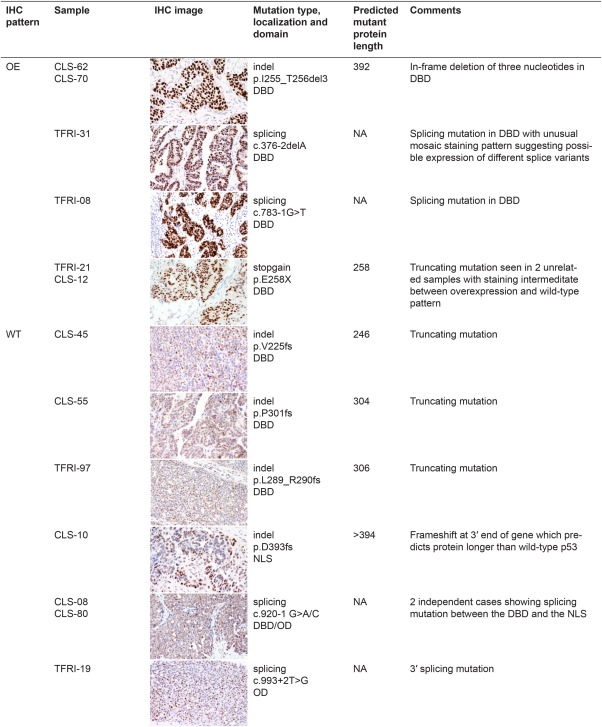
Cases with discordant *TP53* mutation and expected p53 IHC staining pattern.

Comparison of the mutation and staining data provided partial explanations for why p53 protein was detectable (Figures 1a and 5). For example, two unrelated cases had an inframe p.I255del1 mutation that did not alter the reading frame. The observed OE pattern in these cases suggests this mutation may give rise to a nonsynonymous conformational change. Other LOF mutations predicted mutant p53 protein with lengths >250 amino acids (Figure 5).

WT staining was observed in nine HGSOC (5.3%) of which two (1.2%) did not have a detectable *TP53* mutation. Both cases expressed WT1 as evidence of serous cell lineage and showed non‐specific solid architecture and moderate nuclear atypia by morphology (supplementary material, Figure S4).

### Cytoplasmic staining

Cytoplasmic staining (CY) without nuclear overexpression of p53 was observed in four (2.3%) cases of HGSOC and these results were confirmed on full section staining (supplementary material, Figure S5). For two cases, CY staining was also confirmed in independent specimens collected at recurrence. Other nuclear markers (PAX8, WT1, ER) assessed on all four cases did not show any evidence of abnormal staining, excluding the possibility that CY staining for p53 could have arisen artefactually from compromised tissue. The *TP53* mutations in all four cases predicted disruption of the p53 nuclear localization signalling domain located at 316–325 aa.

## Discussion

These results show that use of an optimized p53 IHC assay is an accurate predictor of the presence and class of *TP53* mutations in ovarian carcinoma with high specificity and sensitivity for prediction of GOF mutations and NDM status.

The observed *TP53* mutation frequency for HGSOC cases was 99% compared to 94–97% in previous studies 1, 42. This is likely to be a result of the higher sensitivity of targeted NGS sequencing 9 and improvements in experimental design in this study (including stringent sample selection, manual review of mutation calls and secondary analysis of discordant cases). We observed significant differences in accuracy for the four clinical IHC assays tested (Figure 4). Method 1 had the highest intensity of staining (Figure 3) and had less misclassification. Previous studies have focused on OE as the most important determinant of abnormal p53 staining and p53 IHC has not been optimized for the lower cut‐off needed to distinguish WT from CA. Across the different methods weakly stained WT cases were frequently misclassification as CA causing false positive mutation predictions. Weakly stained assays performed without intrinsic controls cannot reliably distinguish CA from WT. Therefore, we propose that use of intrinsic control cells provides an internal reference for IHC scoring. Despite a common belief that p53 IHC cannot detect p53 wild type protein, the DO7 antibody used in Method 1 robustly detects p53 expression in normal cells including stromal fibroblasts and lymphocytes when used with recent improvements in polymer‐based IHC detection systems. It is possible that p53‐positive intraepithelial lymphocytes in a CA case could be falsely read as p53 WT tumour cells, particularly since some *BRCA1* and *BRCA2* HGSOC can have a very dense intraepithelial lymphocytic infiltrate 43. Yet we believe that a titration towards a stronger staining IHC assay is preferred, not only for better interpretation at the lower cut‐off, but also because of better distinction of OE from high WT cases at the upper cut‐off. Because stronger staining moves that interpretation away from a cut‐off definition by % positive tumour cells towards a pattern interpretation with virtually all tumour cells strongly staining in OE versus the variable intensity with some negative tumour cells seen in WT. Our data strongly support the contention that further assay comparison and training in interpretation are needed for p53 IHC to be used as a diagnostic and predictive test 44.

Our data show that optimized p53 IHC can have 100% specificity for binary classification of pathogenic *TP53* mutation in ovarian carcinoma. This is a remarkable increase compared to 38% reported previously 20. In the prior study, 7/30 cases with OE and 6/17 cases with CA had NDM, which probably resulted from only sequencing exons 4–9 of *TP53* or differences in IHC interpretation. We reduced the number of false positives (cases in which p53 IHC predicts mutation but mutation is absent) for three main reasons: first, we improved identification of *TP53* mutation through sensitive NGS of the entire coding sequence, second, we improved identification of p53 IHC patterns corresponding with the *TP53* mutation by optimized IHC, and third, we improved concordance of both methods by performing a secondary analysis.

In the initial sequencing analysis, we already performed quality control for cases failing sequencing and HGSOC with NDM. We repeated DNA extraction from new cores taken from FFPE blocks. By doing so, we avoided the pitfall of simply not evaluating tumour tissue. This resulted in an additional identification of six *TP53* mutations in eight initially NDM HGSOC in a second round of NGS. Short read NGS, which is thought of having lower sensitivity for indel detection, performed well. Only one splice acceptor mutation (c.356‐2delA) was missed by NGS and only detected by Sanger sequencing (but was CA by IHC and therefore would have been caught in the secondary analysis).

The secondary analysis was performed for NGS‐IHC discordant cases and included DNA re‐extraction, re‐sequencing and full section IHC. After knowing the mutation and p53 IHC status, the interpretation of *TP53* sequencing data was revised in 2/21 cases and p53 IHC data in 5/23 cases. Three EC with NDM showed CA on the tissue microarray cores. We did not consider the intrinsic control in the initial interpretation, which was absent in these cases. On full section, the central areas including the TMA punch holes also showed CA without intrinsic control but there was WT towards the edges of the section. Heterogeneous p53 expression is likely caused by antigen degradation due to delayed fixation of the tissue center. To avoid such pitfalls in interpretation, we recommend that only cases with intrinsic control present should be interpreted. The secondary analysis increased the accuracy of p53 IHC as binary or class predictor of the *TP53* mutation by 3.6% each. These examples illustrate how both methods can complement each other. A discordance forces re‐evalution of both assays. For the most accurate assessment of mutation status currently within the realm of clinical trials, we recommend a combination of NGS and IHC.

Although the secondary analysis resolved issues with discordant GOF cases or NDM cases, it did not improve performance for the LOF mutation class where 13 cases (24%) did not stain as CA. This limits the overall sensitivity of p53 IHC for the binary *TP53* mutational status to 96% and the overall accuracy for the class of mutation to 94.7%, the latter is still higher compared to 83% in a previous study 20. Detailed review of these cases show that the majority have detectable p53 expression owing to 3′ mutations. Stopgains associated with CA occur before amino acid 213 while stopgains associated with WT occur after amino acids 245. Because the DO7 antibody used in method 1 recognizes the N‐terminal region between amino acids 19 and 26 we speculate that early stopgains are subjected to nonsense‐mediated RNA decay while later stopgains are resulting in expression of truncated p53 protein. In other cases indels or splicing mutations resulted in OE. An in frame indel is likely having the same conformational effect as nonsynonymous mutations. In two cases, splice site mutations in direct proximity result in CA and OE. It has been reported that slight changes in location of mutation can have different effects on the alternative splicing process leading to expression of alternative splicing variants 45. P53 IHC is therefore an essential additional method to sequencing to understand the functional effects of *TP53* mutations. p53 IHC further subclassifies LOF into true LOF with CA versus truncating mutations with WT versus putative LOF with OE that may be better classified as GOF.

We have also identified a wider pattern of abnormal p53 expression as we observed CY staining in four (2.3%) of HGSOC with complete interobserver agreement. Since OE cases can show minor amounts of CY it is important to note that CY should only be reported in the absence of strong nuclear expression. Hence, only cases for which the question is WT versus CA that show prominent cytoplasmic staining should be considered for CY. Although CY staining can be artefactual this is an unlikely interpretation of our data as (1) the same p.R306X mutation was detected in two unrelated cases that both showed CY and not in any other case (2), CY was observed in paired primary and recurrence specimens and (3) CY was confirmed on full sections. Cytoplasmic localization of mutated p53 has been reported before in colorectal carcinoma 46. All four mutations associated with CY in our series were indels and stopgains resulting in predicted p53 protein of 292–306 aa length truncated the protein before the nuclear localization domain. However, we observed WT staining for other truncating mutations that resulted in similar protein lengths and there may be alternative mechanisms for cytoplasmic localization. It has been reported that a p.K382fs mutation resulting in a 420 aa protein showed cytoplasmic localization owing to impaired binding to importin. This occurred because of conformational changes from the additional 27 aa and not from any alteration in the nuclear localization domains 47. In addition, specific p53 mutants may undergo post‐translational modifications that can stimulate nuclear transport and/or mitochondrial association, promoting cytoplasmic accumulation. It is important to note that p53 normally shuttles between the nucleus and the cytoplasm, and cytoplasmic functions of p53 are well documented. However, cytoplasmic sequestered p53 cannot exert its nuclear function 48 and CY is likely to be indicative for LOF effects.

For diagnostic pathology, identifying *TP53* status in ovarian carcinoma has critical clinical utility: distinguishing HGSOC from low‐grade serous carcinoma on small tissue biopsies before commencing neoadjuvant chemotherapy 10, identification of STIC 49 and sub‐classification of ovarian carcinomas for inclusion in histotype‐specific clinical trials 19. Our results are transferable to other tumour sites, for example endometrial carcinomas or adenocarcinomas of gastroesophageal junction 50, 51 although the interpretation of p53 IHC may not be straightforward for tumours showing longer periods of terminal differentiation allowing for degradation of nonsynonymously mutated p53 protein and interpretation rules may have to be adjusted 52.

For current clinical practice, which relies mostly on IHC without access to sequencing, a diagnostic limitation for p53 IHC should be kept in mind. Nine (5.3%) of HGSOC in our revised series showed WT staining and 7 (4.1%) cases harboured an underlying LOF mutation. Importantly, this means that the finding of WT p53 IHC, particularly in small biopsies, cannot solely be used to diagnose low‐grade serous tumours. NGS should always be considered in WT IHC with morphological features suggesting HGSOC. However, the major strength of p53 IHC as a clinical test is its high negative predictive value as abnormal p53 IHC virtually excludes the possibility of a low‐grade serous tumour 10.

Only two HGSOC remained wild type by both sequencing and IHC after secondary analysis suggesting that *TP53* wild type HGSOC are rare (≤1%). Some authorities even question a diagnosis of HGSOC if there is no evidence for *TP53* mutation 53. The classification of our wild type HGSOC cases remains uncertain. These tumours represent a rare subset which should be studied to establish whether other mechanisms such as MDM2 amplification can lead to an alternative pathway of HGSOC oncogenesis 1. WT staining in these cases effectively excludes the possibility of homozygous deletion of *TP53*. The prevalence of *TP53* mutations in EC was 8.8% in our series, which is similar to reports for endometrioid carcinomas of the ovary (7%) 54 and endometrium (9%) 55 but remarkably lower compared to 51% (*N* = 37/72) from previous reports that included high‐grade carcinomas, which are now classified as HGSOC 56. This further underscores the importance of accurate disease classification for study inclusion, which we performed using diagnostic IHC marker panels 19.

Our results show that optimized p53 IHC assay, when interpreted correctly, can be a useful surrogate for the *TP53* mutation status. The combination of p53 IHC and sequencing should be considered the gold standard in assessing the p53 functional status for clinical trial inclusion.

## Author Contributions

Conception and design: Köbel M, Brenton JD. Provision of study materials or patients: Köbel M, LePage C, Mes Masson A. Collection and assembly of data: Köbel M, Piskorz AM, Lee S, Liu S, LePage C. Data analysis and Interpretation: Köbel M, Piskorz AM, Marass F, Rosenfeld N, Brenton JD. Manuscript drafting: Köbel M, Piskorz AM, Brenton JD. Manuscript revisions: Köbel M, Piskorz AM, Lee S, Liu S, LePage C, Marass F, Rosenfeld N, Mes Masson A, Brenton JD. Final approval of manuscript: Köbel M, Piskorz AM, Lee S, Liu S, LePage C, Marass F, Rosenfeld N, Mes Masson A, Brenton JD.

## Supporting information

SUPPLEMENTARY MATERIAL ONLINE

**Appendix S1.** Supplementary analytical file

**Appendix S2.** Supplementary analytical file

**Figure S1.** Combined *TP53* mutation detection strategy. All samples were sequenced using tagged‐amplicon sequencing. Sequencing quality control was performed by checking the coverage for each amplicon for two technical replicates and reporting samples and/or *TP53* amplicons with inadequate coverage. Samples and amplicons with low or no coverage had repeat tagged‐amplicon sequencing. Visual inspection with IGV of the indexed BAM files was performed for all variants identified from the bioinformatic analysis to confirm accurate mutation calling. For sequences with ambiguous mutation start and/or end sites further Sanger sequencing was performed. If no mutation was detected by the variant calling algorithms, visual inspection of the whole *TP53* coding region was performed in IGV. If a mutation was still not identifiable then Sanger sequencing was performed

**Figure S2.** Comparison of p53 IHC methods 1 and 4. A, B Low power view on tissue microarray shows stronger staining with method 1 (A) compared to method 4 (B) C, D EC without detectable *TP53* mutation showing wild type pattern with p53 method 1 but CA with method 4 (note lack of internal control). E, F HGSOC with detected nonsynonymous mutation showing overexpression with p53 method 1 but wild type pattern with p53 method 4

**Figure S3.** p53 IHC quality control revisions. (A–C) HGSOC with detected splicing mutation. On tissue microarray (A) interpreted as wild type pattern that was focally present on full section (B) but predominant CA on full section, therefore revised from wild type to CA: note the presence of an internal control (C). (D–F) EC with detected nonsynonymous mutation. On tissue microarray (D) interpreted as wild type that was seen on full section adjacent to tissue microarray core hole (E, black line) but predominant overexpression on full section (F), therefore revised from wild type to overexpression. (G–I) EC without detectable *TP53* mutation. On tissue microarray (G) interpreted as CA despite lack of internal control, also seen on full section adjacent to tissue microarray core hole (I, black line) but areas of wild type towards the edge of full section (I), therefore revised from CA to wild type

**Figure S4.** *TP53* wild type HGSOC. (A, C, E) (COEUR 22) shows a tumour with non‐specific solid architecture (A) and moderate nuclear atypia with evidence of high cell turn over (C) as well as p53 wild type pattern (E) with no evidence of *TP53* mutation by sequencing. This neoplasm expressed WT1 and ARID1A (not shown) and was negative for PAX8 and ER. The patient was 47 years at diagnosis of stage IIIC disease and died 12 months after diagnosis of disease. (B, D, F) (COEUR 100) shows a tumour with solid, vaguely glandular architecture (B), spindled tumour cells with moderate nuclear atypia (D) as well as p53 wild type pattern (F) with no evidence of *TP53* mutation by sequencing. This neoplasm expressed PAX8, WT1, ER and ARID1A (not shown). The patient was 85 years at diagnosis of stage IIIC disease and died 46 months after diagnosis. Both cases showed no evidence of *KRAS, PTEN*, *PIK3CA* or *EGFR* mutation

**Figure S5.** Cytoplasmic staining. (A) HGSOC with non‐artefactual cytoplasmic staining interfering with nuclear interpretation. (B) HGSOC with artefactual cytoplasmic staining at the edge of a core due to technical artefacts

**Table S1.** 18 case selected for initial resequencing

**Table S2.** 23 cases selected for secondary analysis

**Table S3.** Data table

## References

[1] Ahmed AA , Etemadmoghadam D , Temple J , *et al* Driver mutations in TP53 are ubiquitous in high grade serous carcinoma of the ovary. J Pathol 2010; 221: 49–56. 20229506 10.1002/path.2696 PMC3262968

[2] Lee Y , Miron A , Drapkin R , *et al* A candidate precursor to serous carcinoma that originates in the distal fallopian tube. J Pathol 2007; 211: 26–35. 17117391 10.1002/path.2091

[3] Kuhn E , Kurman RJ , Vang R , *et al* TP53 mutations in serous tubal intraepithelial carcinoma and concurrent pelvic high‐grade serous carcinoma‐evidence supporting the clonal relationship of the two lesions. J Pathol 2011; 226: 421–426. 21990067 10.1002/path.3023 PMC4782784

[4] Chien J , Sicotte H , Fan JB , *et al* TP53 mutations, tetraploidy and homologous recombination repair defects in early. Nucleic Acids Res 2015; 43: 6945–6958. 25916844 10.1093/nar/gkv111 PMC4538798

[5] Bashashati A , Ha G , Tone A , *et al* Distinct evolutionary trajectories of primary high‐grade serous ovarian cancers revealed through spatial mutational profiling. J Pathol 2013; 231: 21–34. 23780408 10.1002/path.4230 PMC3864404

[6] Castellarin M , Milne K , Zeng T , *et al* Clonal evolution of high‐grade serous ovarian carcinoma from primary to recurrent disease. J Pathol 2013; 229: 515–524. 22996961 10.1002/path.4105

[7] Schwarz RF , Ng CK , Cooke SL , *et al* Spatial and temporal heterogeneity in high‐grade serous ovarian cancer: a phylogenetic analysis. PLoS Med 2015; 12: e1001789. 25710373 10.1371/journal.pmed.1001789 PMC4339382

[8] Swisher EM , Wollan M , Mahtani SM , *et al* Tumor‐specific p53 sequences in blood and peritoneal fluid of women with. Am J Obstet Gynecol 2005; 193: 662–667. 16150257 10.1016/j.ajog.2005.01.054

[9] Forshew T , Murtaza M , Parkinson C , *et al* Noninvasive identification and monitoring of cancer mutations by targeted deep sequencing of plasma DNA. Sci Transl Med 2012; 4: 136ra68. 10.1126/scitranslmed.3003726 22649089

[10] Altman AD , Nelson GS , Ghatage P , *et al* The diagnostic utility of TP53 and CDKN2A to distinguish ovarian high‐grade serous carcinoma from low‐grade serous ovarian tumors. Mod Pathol 2013; 26: 1255–1263. 23558569 10.1038/modpathol.2013.55

[11] Murtaza M , Dawson S‐J , Tsui DWY , *et al* Non‐invasive analysis of acquired resistance to cancer therapy by sequencing of plasma DNA. Nature 2013; 497: 108–112. 23563269 10.1038/nature12065

[12] McManus DT , Yap EP , Maxwell P , *et al* p53 expression, mutation, and allelic deletion in ovarian cancer. J Pathol 1994; 174: 159–168. 7823248 10.1002/path.1711740304

[13] Casey G , Lopez ME , Ramos JC , *et al* DNA sequence analysis of exons 2 through 11 and immunohistochemical staining are required to detect all known p53 alterations in human malignancies. Oncogene 1996; 13: 1971–1981. 8934544

[14] Leitao MM , Soslow RA , Baergen RN , *et al* Mutation and expression of the TP53 gene in early stage epithelial ovarian carcinoma. Gynecol Oncol 2004; 93: 301–306. 15099937 10.1016/j.ygyno.2004.01.043

[15] Nenutil R , Smardova J , Pavlova S , *et al* Discriminating functional and non‐functional p53 in human tumours by p53 and MDM2 immunohistochemistry. J Pathol 2005; 207: 251–259. 16161005 10.1002/path.1838

[16] Singer G , Stöhr R , Cope L , *et al* Patterns of p53 mutations separate ovarian serous borderline tumors and low‐ and high‐grade carcinomas and provide support for a new model of ovarian carcinogenesis: a mutational analysis with immunohistochemical correlation. Am J Surg Pathol 2005; 29: 218–224. 15644779 10.1097/01.pas.0000146025.91953.8d

[17] de Graeff P , Crijns APG , de Jong S , *et al* Modest effect of p53, EGFR and HER‐2/neu on prognosis in epithelial ovarian cancer: a meta‐analysis. Br J Cancer 2009; 101: 149–159. 19513073 10.1038/sj.bjc.6605112 PMC2713689

[18] Köbel M , Reuss A , du Bois A , *et al* The biological and clinical value of p53 expression in pelvic high‐grade serous carcinomas. J Pathol 2010; 222: 191–198. 20629008 10.1002/path.2744

[19] Köbel M , Rahimi K , Rambau PF , *et al* An Immunohistochemical Algorithm for Ovarian Carcinoma Typing. Int J Gynecol Pathol 2016; 35: 430–441. 26974996 10.1097/PGP.0000000000000274 PMC4978603

[20] Yemelyanova A , Vang R , Kshirsagar M , *et al* Immunohistochemical staining patterns of p53 can serve as a surrogate marker for TP53 mutations in ovarian carcinoma: an immunohistochemical and nucleotide sequencing analysis. Mod Pathol 2011; 24: 1248–1253. 21552211 10.1038/modpathol.2011.85

[21] Muller PAJ , Vousden KH. Mutant p53 in cancer: new functions and therapeutic opportunities. Cancer Cell 2014; 25: 304–317. 24651012 10.1016/j.ccr.2014.01.021 PMC3970583

[22] Bieging KT , Mello SS , Attardi LD. Unravelling mechanisms of p53‐mediated tumour suppression. Nat Rev Cancer 2014; 14: 359–370. 24739573 10.1038/nrc3711 PMC4049238

[23] Zhu J , Sammons MA , Donahue G , *et al* Gain‐of‐function p53 mutants co‐opt chromatin pathways to drive cancer growth. Nature 2015; 525: 206–211. 26331536 10.1038/nature15251 PMC4568559

[24] Olive KP , Tuveson DA , Ruhe ZC , *et al* Mutant p53 gain of function in two mouse models of Li‐Fraumeni syndrome. Cell 2004; 119: 847–860. 15607980 10.1016/j.cell.2004.11.004

[25] Lang GA , Iwakuma T , Suh Y‐A , *et al* Gain of function of a p53 hot spot mutation in a mouse model of Li‐Fraumeni syndrome. Cell 2004; 119: 861–872. 15607981 10.1016/j.cell.2004.11.006

[26] Shahin MS , Hughes JH , Sood AK , *et al* The prognostic significance of p53 tumor suppressor gene alterations in ovarian carcinoma. Cancer 2000; 89: 2006–2017. 11064359 10.1002/1097-0142(20001101)89:9<2006::aid-cncr18>3.3.co;2-z

[27] Wojnarowicz PM , Oros KK , Quinn MCJ , *et al* The genomic landscape of TP53 and p53 annotated high grade ovarian serous carcinomas from a defined founder population associated with patient outcome. PLoS One 2012; 7: e45484. 23029043 10.1371/journal.pone.0045484 PMC3447752

[28] Lehmann S , Bykov VJN , Ali D , *et al* Targeting p53 in vivo: a first‐in‐human study with p53‐targeting compound APR‐246 in refractory hematologic malignancies and prostate cancer. J Clin Oncol 2012; 30: 3633–3639. 22965953 10.1200/JCO.2011.40.7783

[29] Maslon MM , Hupp TR. Drug discovery and mutant p53. Trends Cell Biol 2010; 20: 542–555. 20656489 10.1016/j.tcb.2010.06.005

[30] Wiman KG. Pharmacological reactivation of mutant p53: from protein structure to the cancer patient. Oncogene 2010; 29: 4245–4252. 20498645 10.1038/onc.2010.188

[31] Gourley C , Gabra H , Vergote I , *et al* EUTROC PiSARRO: a phase Ib study combining APR‐246 with standard chemotherapy in platinum sensitive relapsed high grade serous ovarian carcinoma (HGSOC). In ASCO Annual Meeting Proceedings 2015; 33: TPS5605.

[32] Martin L , Grigoryan A , Wang D , *et al* Identification and characterization of small molecules that inhibit nonsense‐mediated RNA decay and suppress nonsense p53 mutations. Cancer Res 2014; 74: 3104–3113. 24662918 10.1158/0008-5472.CAN-13-2235 PMC4040335

[33] Bykov VJN , Wiman KG. Mutant p53 reactivation by small molecules makes its way to the clinic. FEBS Lett 2014; 588: 2622–2627. 24768524 10.1016/j.febslet.2014.04.017

[34] Le Page , Kobel M , de Ladurantaye , *et al* Specimen quality evaluation in Canadian biobanks participating in the COEUR. Biopreserv Biobank 2013; 11: 83–93. 24845429 10.1089/bio.2012.0044

[35] Bromley AB , Altman AD , Chu P , *et al* Architectural patterns of ovarian/pelvic high‐grade serous carcinoma. Int J Gynecol Pathol 2012; 31: 397–404. 22833078 10.1097/PGP.0b013e31824c2372

[36] Thorvaldsdottir H , Robinson JT , Mesirov JP. Integrative Genomics Viewer (IGV): high‐performance genomics data visualization. Briefing Bioinform 2013; 14: 178–192. 10.1093/bib/bbs017 PMC3603213 22517427

[37] Sjoblom T , Jones S , Wood LD , *et al* The consensus coding sequences of human breast and colorectal cancers. Science 2006; 314: 268–274. 16959974 10.1126/science.1133427

[38] Barber BR , Biron VL , Klimowicz AC , *et al* Molecular predictors of locoregional and distant metastases in oropharyngeal squamous cell carcinoma. J Otolaryngol Head Neck Surg 2013; 42: 53. 24401183 10.1186/1916-0216-42-53 PMC3819019

[39] R Core Team (2015). R: a language and environment for statistical computing. R Foundation for Statistical Computing, Vienna, Austria. [Accessed 1 September 2015]: available from: https://www.R-project.org/

[40] Caret: Classification and Regression Training. R package version 6.0‐52. [Accessed 1 September 2015]: available from: https://cran.r-project.org/web/packages/caret/index.html

[41] Stodden V , Leisch F , Peng RD. Implementing Reproducible Research. Chapman and Hall/CRC London, UK. 2014; 1–448.

[42] Bell D , Berchuck A , Birrer M , *et al* Integrated genomic analyses of ovarian carcinoma. Nature 2011; 474: 609–615. 21720365 10.1038/nature10166 PMC3163504

[43] McAlpine JN , Porter H , Köbel M , *et al* BRCA1 and BRCA2 mutations correlate with TP53 abnormalities and presence of immune cell infiltrates in ovarian high‐grade serous carcinoma. Mod Pathol 2012; 25: 740–750. 22282309 10.1038/modpathol.2011.211

[44] Lee S , Piskorz AM , Le Page C , *et al* Calibration and optimization of p53, WT1, and Napsin A immunohistochemistry ancillary tests for histotyping of ovarian carcinoma: Canadian Immunohistochemistry Quality Control (CIQC) experience. Int J Gynecol Pathol 2016; 35: 209–221. 26598982 10.1097/PGP.0000000000000251

[45] Cooks T , Pateras IS , Tarcic O , *et al* Mutant p53 prolongs NF‐kappaB activation and promotes chronic inflammation and. Cancer Cell 2013; 23: 634–646. 23680148 10.1016/j.ccr.2013.03.022 PMC3657134

[46] Jansson A , Gentile M , Sun XF. p53 Mutations are present in colorectal cancer with cytoplasmic p53 accumulation. Int J Cancer 2001; 92: 338–341. 11291068 10.1002/ijc.1189

[47] Komlodi‐Pasztor E , Trostel S , Sackett D , *et al* Impaired p53 binding to importin: a novel mechanism of cytoplasmic sequestration identified in oxaliplatin‐resistant cells. Oncogene 2009; 28: 3111–3120. 19581934 10.1038/onc.2009.166 PMC2867326

[48] Green DR , Kroemer G. Cytoplasmic functions of the tumour suppressor p53. Nature 2009; 458: 1127–1130. 19407794 10.1038/nature07986 PMC2814168

[49] Visvanathan K , Vang R , Shaw P , *et al* Diagnosis of serous tubal intraepithelial carcinoma based on morphologic and immunohistochemical features: a reproducibility study. Am J Surg Pathol 2011; 35: 1766–1775. 21989347 10.1097/PAS.0b013e31822f58bc PMC4612640

[50] Han G , Sidhu D , Duggan MA , *et al* Reproducibility of histological cell type in high‐grade endometrial carcinoma. Mod Pathol 2013; 26: 1594–1604. 23807777 10.1038/modpathol.2013.102

[51] Kaye PV , Haider SA , James PD , *et al* Novel staining pattern of p53 in Barrett's dysplasia – the absent pattern. Histopathology 2010; 57: 933–935. 21166706 10.1111/j.1365-2559.2010.03715.x

[52] Singh N , Leen SL , Han G , *et al* Expanding the morphologic spectrum of differentiated VIN (dVIN) through detailed mapping of cases with p53 loss. Am J Surg Pathol 2015; 39: 52–60. 25025443 10.1097/PAS.0000000000000291

[53] Vang R , Levine DA , Soslow RA , *et al* Molecular Alterations of TP53 are a defining feature of ovarian high‐grade serous carcinoma: a rereview of cases lacking TP53 mutations in the cancer genome atlas ovarian study. Int J Gynecol Pathol 2015; 35: 48–55. 10.1097/PGP.0000000000000207 PMC4696053 26166714

[54] McConechy MK , Ding J , Senz J , *et al* Ovarian and endometrial endometrioid carcinomas have distinct CTNNB1 and PTEN mutation profiles. Mod Pathol 2013; 27: 128–134. 23765252 10.1038/modpathol.2013.107 PMC3915240

[55] Kandoth C , Schultz N , Cherniack AD , *et al* Integrated genomic characterization of endometrial carcinoma. Nature 2013; 497: 67–73. 23636398 10.1038/nature12113 PMC3704730

[56] Wu R , Hendrix‐Lucas N , Kuick R , *et al* Mouse model of human ovarian endometrioid adenocarcinoma based on somatic defects in the Wnt/beta‐catenin and PI3K/Pten signaling pathways. Cancer Cell 2007; 11: 321–333. 17418409 10.1016/j.ccr.2007.02.016

