# Supplementary material for: Optimized p53 immunohistochemistry is an accurate predictor of TP53 mutation in ovarian carcinoma
Source: J Pathol Clin Res. 2016 Jul 13;2(4):247–58. doi: 10.1002/cjp2.53 (PMC5091634; doi:10.1002/cjp2.53)
Supplement: Supplementary file 1 — Appendix S1. Supplementary analytical file [file CJP2-2-247-s001.docx]

# Koebel et al TP53 MS Data Analysis

## Data

This is an R Markdown document describing the statistical analysis of Koebel et al (2016). Required libraries are loaded. We use Microsoft R Open 3.2.5 with the snapshot of CRAN packages on 1 May 2016.

##
## checkpoint: Part of the Reproducible R Toolkit from Microsoft
## https://mran.microsoft.com/documents/rro/reproducibility/

## Scanning for packages used in this project

## Warning in deps.R(tempfile): No file at path 'C:\Users\brento01\AppData
## \Local\Temp\RtmpCisqmt\file8f467bd5370.Rmd'.

## Warning in deps.R(tempfile): No file at path 'C:\Users\brento01\AppData
## \Local\Temp\RtmpCisqmt\file8f46e4622ed.Rmd'.

## - Discovered 5 packages

## Unable to parse 2 files:

## - Koebel_MS_analysis.Rmd

## - SEARCH_TP53.Rmd

## All detected packages already installed

## checkpoint process complete

## ---

## Loading required package: readr

## Loading required package: tidyr

## Loading required package: knitr

## Loading required package: dplyr

##
## Attaching package: 'dplyr'

## The following objects are masked from 'package:stats':
##
## filter, lag

## The following objects are masked from 'package:base':
##
## intersect, setdiff, setequal, union

## Loading required package: ggplot2

## Loading required package: irr

## Loading required package: lpSolve

## Loading required package: caret

## Loading required package: lattice

## Loading required package: e1071

## Loading required package: grid

We read in the tab delimited file containing the sequencing and immunohistochemistry results into a data frame, convert integer identifiers and scores to factors, add additional indicator variables and print out the structure of the data.

## Cohort Sample.Name Histotype Exclusion.QC Exclusion.Reason
## 1 COEUR ATiM_0903 HGSOC FAIL poor seq quality
## 2 COEUR ATiM_0929 EC FAIL poor seq quality
## 3 COEUR ATiM_0478 HGSOC FAIL poor seq quality
## 4 COEUR ATiM_0441 EC PASS no additional DNA available
## 5 COEUR ATiM_0414 HGSOC FAIL poor seq quality
## 6 CLS CLS_46 HGSOC FAIL poor seq quality
## 7 COEUR TFRI-051 HGSOC FAIL poor seq quality
## 8 COEUR TFRI-088 HGSOC PASS no additional DNA available
## IHC.method.1 IHC.method.2 IHC.method.3 IHC.method.4
## 1 1 1 1 1
## 2 1 0 0 1
## 3 2 2 2 2
## 4 1 1 1 1
## 5 2 NA NA NA
## 6 2 2 2 2
## 7 2 2 1 1
## 8 2 2 NA 2

## Experimental design

Summarize numbers of cases by histotype (EC, endometrioid ovarian carcinoma; HGSOC, high-grade serous ovarian carcinoma).

## Source: local data frame [2 x 3]
##
## Histotype n freq
## (fctr) (int) (dbl)
## 1 EC 80 31.87
## 2 HGSOC 171 68.13

Summarize age by histotype.

## Source: local data frame [2 x 5]
##
## Histotype Median_Age IQR_Age Age_Q1 Age_Q3
## (fctr) (dbl) (dbl) (dbl) (dbl)
## 1 EC 55 16.75 49 65.75
## 2 HGSOC 58 15.00 52 67.00

Summarize by stage by histotype.

## Source: local data frame [10 x 4]
## Groups: Histotype [2]
##
## Histotype Stage.bin n freq
## (fctr) (chr) (int) (dbl)
## 1 EC I 50 62.500
## 2 EC II 19 23.750
## 3 EC III 6 7.500
## 4 EC IV 2 2.500
## 5 EC unknown 3 3.750
## 6 HGSOC I 7 4.094
## 7 HGSOC II 7 4.094
## 8 HGSOC III 113 66.082
## 9 HGSOC IV 31 18.129
## 10 HGSOC unknown 13 7.602

### Analysis of Sequencing Results

Estimate median sequencing depth and allele fraction for TP53 sequencing.

## Seq.Depth Allele.Fraction
## Min. : 28 Min. :0.01
## 1st Qu.: 2000 1st Qu.:0.44
## Median : 3635 Median :0.65
## Mean : 4539 Mean :0.60
## 3rd Qu.: 6582 3rd Qu.:0.79
## Max. :15002 Max. :0.96
## NA's :79 NA's :79


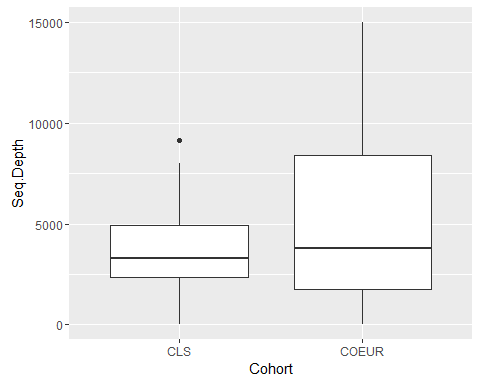

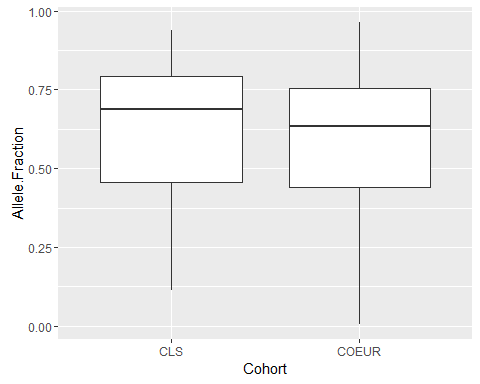


Summarize results for type of TP53 mutation for all cases.


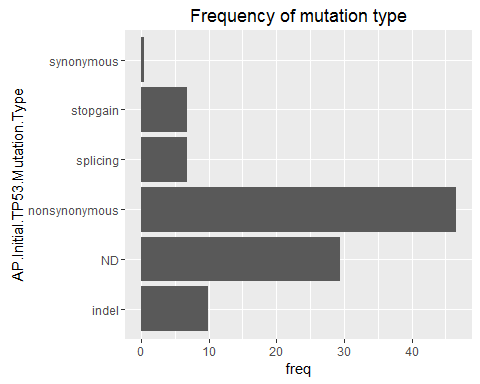


Summarize results for type of mutation.

| Histotype | AP.Initial.TP53.Mutation.Type | n | freq |
| --- | --- | --- | --- |
| EC | indel | 2 | 2.50 |
| EC | ND | 72 | 90.00 |
| EC | nonsynonymous | 5 | 6.25 |
| EC | synonymous | 1 | 1.25 |
| HGSOC | indel | 23 | 13.45 |
| HGSOC | ND | 2 | 1.17 |
| HGSOC | nonsynonymous | 112 | 65.50 |
| HGSOC | splicing | 17 | 9.94 |
| HGSOC | stopgain | 17 | 9.94 |


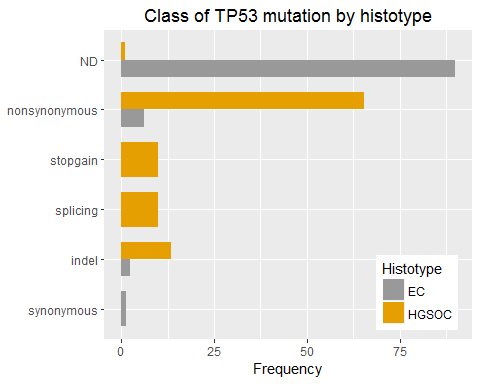


Replot as Cleveland plot.


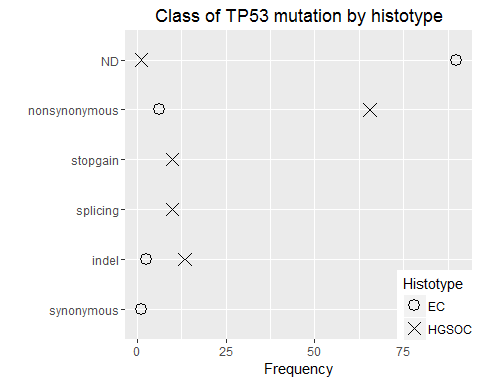


Which was the commonest non-synonymous change across all cases?

## Source: local data frame [113 x 3]
##
## protein.Effect n freq
## (chr) (int) (dbl)
## 1 NA 77 30.677
## 2 p.? 16 6.375
## 3 p.R175H 9 3.586
## 4 p.Y220C 7 2.789
## 5 p.R273H 5 1.992
## 6 p.R196X 4 1.594
## 7 p.G245C 3 1.195
## 8 p.H193R 3 1.195
## 9 p.R213X 3 1.195
## 10 p.R248Q 3 1.195
## .. ... ... ...

Which was the commonest non-synonymous change in HGSOC cases?

## Source: local data frame [110 x 3]
##
## protein.Effect n freq
## (chr) (int) (dbl)
## 1 p.? 16 9.357
## 2 p.R175H 9 5.263
## 3 p.Y220C 6 3.509
## 4 p.R273H 5 2.924
## 5 p.R196X 4 2.339
## 6 p.G245C 3 1.754
## 7 p.H193R 3 1.754
## 8 p.R213X 3 1.754
## 9 p.R248Q 3 1.754
## 10 p.R248W 3 1.754
## .. ... ... ...

## Immunohistochemical staining for p53

We first compare the performance of the 4 different p53 immunohistochemical (IHC) methods. The expression of p53 was scored in a 4-tier system: complete absence, wild type pattern (nuclear staining of variable intensity from 1-79% of tumour cells), overexpression (>80% of tumour cells with strong nuclear staining), and cytoplasmic staining. These correspond to scores of 0-3 respectively.

We summarize the number of cores stained by histotype and method.

## Histotype Method Score n Freq
## 1 EC IHC.method.1 0 4 5.0000
## 2 EC IHC.method.1 1 73 91.2500
## 3 EC IHC.method.1 2 3 3.7500
## 4 EC IHC.method.2 0 13 16.2500
## 5 EC IHC.method.2 1 64 80.0000
## 6 EC IHC.method.2 2 2 2.5000
## 7 EC IHC.method.2 Unscored 1 1.2500
## 8 EC IHC.method.3 0 17 21.2500
## 9 EC IHC.method.3 1 59 73.7500
## 10 EC IHC.method.3 2 2 2.5000
## 11 EC IHC.method.3 Unscored 2 2.5000
## 12 EC IHC.method.4 0 21 26.2500
## 13 EC IHC.method.4 1 55 68.7500
## 14 EC IHC.method.4 2 2 2.5000
## 15 EC IHC.method.4 Unscored 2 2.5000
## 16 HGSOC IHC.method.1 0 39 22.8070
## 17 HGSOC IHC.method.1 1 10 5.8480
## 18 HGSOC IHC.method.1 2 118 69.0058
## 19 HGSOC IHC.method.1 3 3 1.7544
## 20 HGSOC IHC.method.1 Unscored 1 0.5848
## 21 HGSOC IHC.method.2 0 44 25.7310
## 22 HGSOC IHC.method.2 1 6 3.5088
## 23 HGSOC IHC.method.2 2 109 63.7427
## 24 HGSOC IHC.method.2 3 1 0.5848
## 25 HGSOC IHC.method.2 Unscored 11 6.4327
## 26 HGSOC IHC.method.3 0 42 24.5614
## 27 HGSOC IHC.method.3 1 12 7.0175
## 28 HGSOC IHC.method.3 2 94 54.9708
## 29 HGSOC IHC.method.3 Unscored 23 13.4503
## 30 HGSOC IHC.method.4 0 45 26.3158
## 31 HGSOC IHC.method.4 1 19 11.1111
## 32 HGSOC IHC.method.4 2 97 56.7251
## 33 HGSOC IHC.method.4 3 1 0.5848
## 34 HGSOC IHC.method.4 Unscored 9 5.2632

## Scale for 'fill' is already present. Adding another scale for 'fill',
## which will replace the existing scale.


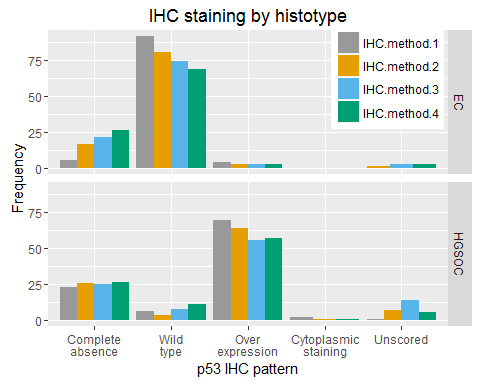


Replot as Cleveland plot.


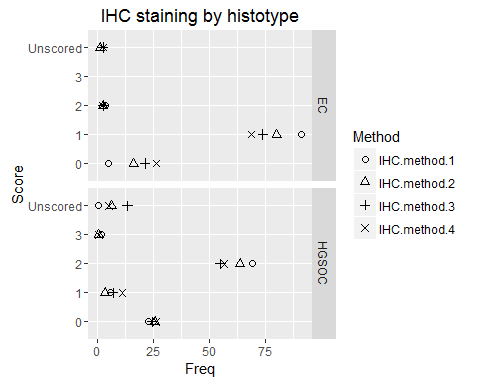


We table frequency for p53 scores for HGSOC and EC cases.

| Histotype | Score | IHC.method.1 | IHC.method.2 | IHC.method.3 | IHC.method.4 |
| --- | --- | --- | --- | --- | --- |
| EC | 0 | 5 | 16 | 21 | 26 |
| EC | 1 | 91 | 80 | 74 | 69 |
| EC | 2 | 4 | 2 | 2 | 2 |
| EC | Unscored | NA | 1 | 2 | 2 |
| HGSOC | 0 | 23 | 26 | 25 | 26 |
| HGSOC | 1 | 6 | 4 | 7 | 11 |
| HGSOC | 2 | 69 | 64 | 55 | 57 |
| HGSOC | 3 | 2 | 1 | NA | 1 |
| HGSOC | Unscored | 1 | 6 | 13 | 5 |

## Inter-rater variability for IHC scoring

Scores from a second, independent observer for a subset of cases stained using method 1

## .
## 0 1 2 3 <NA>
## 27 69 50 3 102

The concordance between obs1 and obs2 for the same cases is shown in a contingency table.

## IHC.method.1.obs2
## IHC.method.1 0 1 2 3
## 0 19 2 0 0
## 1 8 67 3 0
## 2 0 0 47 0
## 3 0 0 0 2

Most disagreement is between "complete absence" (score 0) and "wild type pattern" (score 1) staining. We use Cohen's Kappa to check for inter rater variability for IHC scoring between obs1 and obs2. Weighting with equal weights is used to penalize disagreement (e.g. a score of 0 should not be confused with a score of 2 or 3).

## Cohen's Kappa for 2 Raters (Weights: equal)
##
## Subjects = 148
## Raters = 2
## Kappa = 0.883
##
## z = 14.7
## p-value = 0

## Concordance of TP53 mutation status with p53 IHC

### Binary and ternary class prediction

How many are evaluable for comparison across all IHC methods?

## Source: local data frame [2 x 2]
##
## Histotype n
## (fctr) (int)
## 1 EC 76
## 2 HGSOC 147

We construct a confusion matrix for each IHC staining method comparing (1) binary classification of any abnormal staining to abnormal mutation and (2) prediction of the ternary class of TP53 mutation by IHC.

## The following `from` values were not present in `x`: 3
## The following `from` values were not present in `x`: 3

Extract results for binary class prediction using Method 1-4 and Revised Method 1.

Binary prediction using different IHC Methods

| Prediction.data | Sensitivity | Specificity | Pos Pred Value | Neg Pred Value | Prevalence | Detection Rate | Detection Prevalence | Balanced Accuracy | Mutation.class | IHC.method |
| --- | --- | --- | --- | --- | --- | --- | --- | --- | --- | --- |
| Method 1 (Binary prediction) | 0.931 | 0.947 | 0.976 | 0.855 | 0.700 | 0.652 | 0.668 | 0.939 | Abnormal | 1 |
| Method 2 (Binary prediction) | 0.945 | 0.824 | 0.923 | 0.871 | 0.690 | 0.653 | 0.707 | 0.885 | Abnormal | 2 |
| Method 3 (Binary prediction) | 0.903 | 0.778 | 0.897 | 0.789 | 0.681 | 0.615 | 0.686 | 0.840 | Abnormal | 3 |
| Method 4 (Binary prediction) | 0.874 | 0.726 | 0.880 | 0.716 | 0.696 | 0.608 | 0.692 | 0.800 | Abnormal | 4 |
| Revised Method 1 (Binary prediction) | 0.960 | 1.000 | 1.000 | 0.916 | 0.695 | 0.667 | 0.667 | 0.980 | Abnormal | 1 Revised |

Extract results for ternary class prediction using Method 1-4 and Revised Method 1.

Ternary prediction using different IHC methods

| Prediction.data | Sensitivity | Specificity | Pos.Pred.Value | Neg.Pred.Value | Prevalence | Detection.Rate | Detection.Prevalence | Balanced.Accuracy | Mutation.class | IHC.method |
| --- | --- | --- | --- | --- | --- | --- | --- | --- | --- | --- |
| Method 1 (Ternary prediction) | 0.97 | 0.95 | 0.94 | 0.98 | 0.47 | 0.46 | 0.49 | 0.96 | Gain of function | 1 |
| Method 1 (Ternary prediction) | 0.73 | 0.98 | 0.93 | 0.93 | 0.22 | 0.16 | 0.17 | 0.86 | Loss of function | 1 |
| Method 1 (Ternary prediction) | 0.95 | 0.93 | 0.86 | 0.98 | 0.30 | 0.29 | 0.34 | 0.94 | Wild-type | 1 |
| Method 2 (Ternary prediction) | 0.95 | 0.95 | 0.95 | 0.95 | 0.47 | 0.44 | 0.47 | 0.95 | Gain of function | 2 |
| Method 2 (Ternary prediction) | 0.83 | 0.93 | 0.77 | 0.95 | 0.22 | 0.18 | 0.24 | 0.88 | Loss of function | 2 |
| Method 2 (Ternary prediction) | 0.82 | 0.95 | 0.87 | 0.92 | 0.31 | 0.26 | 0.29 | 0.88 | Wild-type | 2 |
| Method 3 (Ternary prediction) | 0.90 | 0.98 | 0.97 | 0.92 | 0.46 | 0.41 | 0.42 | 0.94 | Gain of function | 3 |
| Method 3 (Ternary prediction) | 0.84 | 0.91 | 0.73 | 0.95 | 0.23 | 0.19 | 0.26 | 0.88 | Loss of function | 3 |
| Method 3 (Ternary prediction) | 0.78 | 0.90 | 0.79 | 0.90 | 0.32 | 0.25 | 0.31 | 0.84 | Wild-type | 3 |
| Method 4 (Ternary prediction) | 0.85 | 0.97 | 0.96 | 0.88 | 0.47 | 0.40 | 0.41 | 0.91 | Gain of function | 4 |
| Method 4 (Ternary prediction) | 0.83 | 0.89 | 0.68 | 0.95 | 0.23 | 0.19 | 0.28 | 0.86 | Loss of function | 4 |
| Method 4 (Ternary prediction) | 0.73 | 0.87 | 0.72 | 0.88 | 0.31 | 0.22 | 0.31 | 0.80 | Wild-type | 4 |
| Revised Method 1 (Ternary prediction) | 1.00 | 0.95 | 0.95 | 1.00 | 0.47 | 0.47 | 0.49 | 0.98 | Gain of function | 1 Revised |
| Revised Method 1 (Ternary prediction) | 0.76 | 1.00 | 1.00 | 0.94 | 0.22 | 0.17 | 0.17 | 0.88 | Loss of function | 1 Revised |
| Revised Method 1 (Ternary prediction) | 1.00 | 0.96 | 0.92 | 1.00 | 0.31 | 0.31 | 0.34 | 0.98 | Wild-type | 1 Revised |

Plot sensitivity and specificity as Cleveland plot to compare performance.


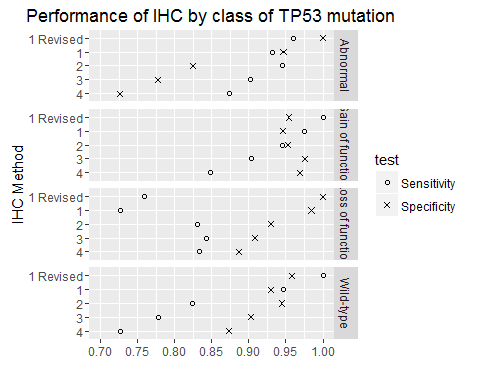


We graph the accuracy of each test with 95% confidence intervals.


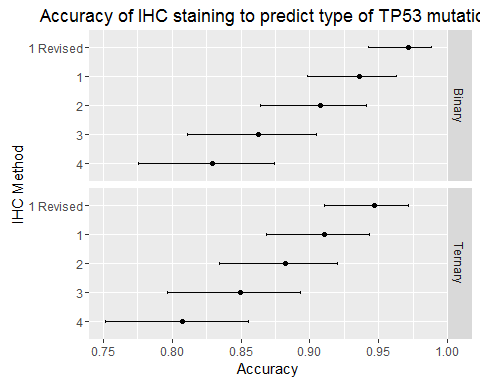


How did Method 1 perform before secondary analysis? We show the data for the binary and ternary comparisons before and after revision for the discordant cases.

## $`Method 1 (Binary prediction)`
## Reference
## Prediction abnormal wt
## abnormal 163 4
## wt 12 71
##
## $`Revised Method 1 (Binary prediction)`
## Reference
## Prediction abnormal wt
## abnormal 166 0
## wt 7 76
##
## $`Method 1 (Ternary prediction)`
## Reference
## Prediction gof lof wt
## gof 114 6 1
## lof 0 40 3
## wt 3 9 71
##
## $`Revised Method 1 (Ternary prediction)`
## Reference
## Prediction gof lof wt
## gof 115 6 0
## lof 0 41 0
## wt 0 7 76

How many HGSOC cases were scored as WT?

| IHC.method.1 | IHC.method.1.obs2 | IHC.method.1.revised | AP.Initial.TP53.Mutation.Type | AP.Final.TP53.Mutation.Type |
| --- | --- | --- | --- | --- |
| 1 | 1 | 1 | splicing | splicing |
| 1 | 1 | 1 | indel | indel |
| 1 | 0 | 0 | splicing | splicing |
| 1 | 1 | 1 | indel | indel |
| 1 | 2 | 1 | indel | indel |
| 1 | 1 | 1 | splicing | splicing |
| 1 | NA | 1 | nonsynonymous | ND |
| 1 | NA | 1 | splicing | splicing |
| 1 | NA | 1 | ND | ND |
| 1 | NA | 1 | indel | indel |

List all the revised IHC cases based on restaining of original tumour block. Note that cytoplasmic staining was investigated separately.

| Histotype | Method1 | Method1Rev | Method1.obs2 | IHC.Block | IHC.blockCY | Initial.Mut.Type | Rev.Mut.Type |
| --- | --- | --- | --- | --- | --- | --- | --- |
| EC | 1 | 2 | 1 | 2 | 2 | nonsynonymous | nonsynonymous |
| EC | 0 | 1 | 0 | 1 | 1 | ND | ND |
| EC | 0 | 1 | 0 | 1 | 1 | ND | ND |
| EC | 0 | 1 | 0 | 1 | 1 | ND | ND |
| HGSOC | 1 | 0 | 0 | 0 | 0 | splicing | splicing |

Print contingency tables by binary and ternary IHC classification against type of mutation.

## TP53.mut.rev
## Method.1.revised_binaryIHC indel ND nonsynonymous splicing stopgain
## abnormal 20 0 115 14 17
## wt 4 75 0 3 0
## Sum 24 75 115 17 17
## TP53.mut.rev
## Method.1.revised_binaryIHC synonymous Sum
## abnormal 0 166
## wt 1 83
## Sum 1 249

## TP53.mut.rev
## Method.1.revised_ternaryIHC indel ND nonsynonymous splicing stopgain
## gof 2 0 115 2 2
## lof 16 0 0 12 13
## wt 4 75 0 3 0
## Sum 22 75 115 17 15
## TP53.mut.rev
## Method.1.revised_ternaryIHC synonymous Sum
## gof 0 121
## lof 0 41
## wt 1 83
## Sum 1 245

Print out complete confusion table data.

## $`Method 1 (Binary prediction)`
## Confusion Matrix and Statistics
##
## Reference
## Prediction abnormal wt
## abnormal 163 4
## wt 12 71
##
## Accuracy : 0.936
## 95% CI : (0.898, 0.963)
## No Information Rate : 0.7
## P-Value [Acc > NIR] : <2e-16
##
## Kappa : 0.852
## Mcnemar's Test P-Value : 0.0801
##
## Sensitivity : 0.931
## Specificity : 0.947
## Pos Pred Value : 0.976
## Neg Pred Value : 0.855
## Prevalence : 0.700
## Detection Rate : 0.652
## Detection Prevalence : 0.668
## Balanced Accuracy : 0.939
##
## 'Positive' Class : abnormal
##
##
## $`Method 2 (Binary prediction)`
## Confusion Matrix and Statistics
##
## Reference
## Prediction abnormal wt
## abnormal 156 13
## wt 9 61
##
## Accuracy : 0.908
## 95% CI : (0.864, 0.941)
## No Information Rate : 0.69
## P-Value [Acc > NIR] : 6.76e-16
##
## Kappa : 0.781
## Mcnemar's Test P-Value : 0.522
##
## Sensitivity : 0.945
## Specificity : 0.824
## Pos Pred Value : 0.923
## Neg Pred Value : 0.871
## Prevalence : 0.690
## Detection Rate : 0.653
## Detection Prevalence : 0.707
## Balanced Accuracy : 0.885
##
## 'Positive' Class : abnormal
##
##
## $`Method 3 (Binary prediction)`
## Confusion Matrix and Statistics
##
## Reference
## Prediction abnormal wt
## abnormal 139 16
## wt 15 56
##
## Accuracy : 0.863
## 95% CI : (0.811, 0.905)
## No Information Rate : 0.681
## P-Value [Acc > NIR] : 2.61e-10
##
## Kappa : 0.683
## Mcnemar's Test P-Value : 1
##
## Sensitivity : 0.903
## Specificity : 0.778
## Pos Pred Value : 0.897
## Neg Pred Value : 0.789
## Prevalence : 0.681
## Detection Rate : 0.615
## Detection Prevalence : 0.686
## Balanced Accuracy : 0.840
##
## 'Positive' Class : abnormal
##
##
## $`Method 4 (Binary prediction)`
## Confusion Matrix and Statistics
##
## Reference
## Prediction abnormal wt
## abnormal 146 20
## wt 21 53
##
## Accuracy : 0.829
## 95% CI : (0.775, 0.875)
## No Information Rate : 0.696
## P-Value [Acc > NIR] : 1.66e-06
##
## Kappa : 0.598
## Mcnemar's Test P-Value : 1
##
## Sensitivity : 0.874
## Specificity : 0.726
## Pos Pred Value : 0.880
## Neg Pred Value : 0.716
## Prevalence : 0.696
## Detection Rate : 0.608
## Detection Prevalence : 0.692
## Balanced Accuracy : 0.800
##
## 'Positive' Class : abnormal
##
##
## $`Revised Method 1 (Binary prediction)`
## Confusion Matrix and Statistics
##
## Reference
## Prediction abnormal wt
## abnormal 166 0
## wt 7 76
##
## Accuracy : 0.972
## 95% CI : (0.943, 0.989)
## No Information Rate : 0.695
## P-Value [Acc > NIR] : <2e-16
##
## Kappa : 0.935
## Mcnemar's Test P-Value : 0.0233
##
## Sensitivity : 0.960
## Specificity : 1.000
## Pos Pred Value : 1.000
## Neg Pred Value : 0.916
## Prevalence : 0.695
## Detection Rate : 0.667
## Detection Prevalence : 0.667
## Balanced Accuracy : 0.980
##
## 'Positive' Class : abnormal
##
##
## $`Method 1 (Ternary prediction)`
## Confusion Matrix and Statistics
##
## Reference
## Prediction gof lof wt
## gof 114 6 1
## lof 0 40 3
## wt 3 9 71
##
## Overall Statistics
##
## Accuracy : 0.911
## 95% CI : (0.868, 0.943)
## No Information Rate : 0.474
## P-Value [Acc > NIR] : <2e-16
##
## Kappa : 0.858
## Mcnemar's Test P-Value : 0.0186
##
## Statistics by Class:
##
## Class: gof Class: lof Class: wt
## Sensitivity 0.974 0.727 0.947
## Specificity 0.946 0.984 0.930
## Pos Pred Value 0.942 0.930 0.855
## Neg Pred Value 0.976 0.926 0.976
## Prevalence 0.474 0.223 0.304
## Detection Rate 0.462 0.162 0.287
## Detection Prevalence 0.490 0.174 0.336
## Balanced Accuracy 0.960 0.856 0.938
##
## $`Method 2 (Ternary prediction)`
## Confusion Matrix and Statistics
##
## Reference
## Prediction gof lof wt
## gof 105 5 1
## lof 1 44 12
## wt 5 4 61
##
## Overall Statistics
##
## Accuracy : 0.882
## 95% CI : (0.834, 0.92)
## No Information Rate : 0.466
## P-Value [Acc > NIR] : <2e-16
##
## Kappa : 0.816
## Mcnemar's Test P-Value : 0.0252
##
## Statistics by Class:
##
## Class: gof Class: lof Class: wt
## Sensitivity 0.946 0.830 0.824
## Specificity 0.953 0.930 0.945
## Pos Pred Value 0.946 0.772 0.871
## Neg Pred Value 0.953 0.950 0.923
## Prevalence 0.466 0.223 0.311
## Detection Rate 0.441 0.185 0.256
## Detection Prevalence 0.466 0.239 0.294
## Balanced Accuracy 0.949 0.880 0.885
##
## $`Method 3 (Ternary prediction)`
## Confusion Matrix and Statistics
##
## Reference
## Prediction gof lof wt
## gof 93 3 0
## lof 0 43 16
## wt 10 5 56
##
## Overall Statistics
##
## Accuracy : 0.85
## 95% CI : (0.796, 0.894)
## No Information Rate : 0.456
## P-Value [Acc > NIR] : < 2e-16
##
## Kappa : 0.768
## Mcnemar's Test P-Value : 0.000306
##
## Statistics by Class:
##
## Class: gof Class: lof Class: wt
## Sensitivity 0.903 0.843 0.778
## Specificity 0.976 0.909 0.903
## Pos Pred Value 0.969 0.729 0.789
## Neg Pred Value 0.923 0.952 0.897
## Prevalence 0.456 0.226 0.319
## Detection Rate 0.412 0.190 0.248
## Detection Prevalence 0.425 0.261 0.314
## Balanced Accuracy 0.939 0.876 0.840
##
## $`Method 4 (Ternary prediction)`
## Confusion Matrix and Statistics
##
## Reference
## Prediction gof lof wt
## gof 95 3 1
## lof 2 45 19
## wt 15 6 53
##
## Overall Statistics
##
## Accuracy : 0.808
## 95% CI : (0.752, 0.856)
## No Information Rate : 0.469
## P-Value [Acc > NIR] : < 2e-16
##
## Kappa : 0.703
## Mcnemar's Test P-Value : 0.000247
##
## Statistics by Class:
##
## Class: gof Class: lof Class: wt
## Sensitivity 0.848 0.833 0.726
## Specificity 0.969 0.886 0.873
## Pos Pred Value 0.960 0.682 0.716
## Neg Pred Value 0.879 0.948 0.879
## Prevalence 0.469 0.226 0.305
## Detection Rate 0.397 0.188 0.222
## Detection Prevalence 0.414 0.276 0.310
## Balanced Accuracy 0.908 0.860 0.800
##
## $`Revised Method 1 (Ternary prediction)`
## Confusion Matrix and Statistics
##
## Reference
## Prediction gof lof wt
## gof 115 6 0
## lof 0 41 0
## wt 0 7 76
##
## Overall Statistics
##
## Accuracy : 0.947
## 95% CI : (0.911, 0.971)
## No Information Rate : 0.469
## P-Value [Acc > NIR] : <2e-16
##
## Kappa : 0.915
## Mcnemar's Test P-Value : NA
##
## Statistics by Class:
##
## Class: gof Class: lof Class: wt
## Sensitivity 1.000 0.759 1.000
## Specificity 0.954 1.000 0.959
## Pos Pred Value 0.950 1.000 0.916
## Neg Pred Value 1.000 0.936 1.000
## Prevalence 0.469 0.220 0.310
## Detection Rate 0.469 0.167 0.310
## Detection Prevalence 0.494 0.167 0.339
## Balanced Accuracy 0.977 0.880 0.979

## Session Information

Print session info.

sessionInfo()

## R version 3.2.5 (2016-04-14)
## Platform: x86_64-w64-mingw32/x64 (64-bit)
## Running under: Windows 7 x64 (build 7601) Service Pack 1
##
## locale:
## [1] LC_COLLATE=English_United Kingdom.1252
## [2] LC_CTYPE=English_United Kingdom.1252
## [3] LC_MONETARY=English_United Kingdom.1252
## [4] LC_NUMERIC=C
## [5] LC_TIME=English_United Kingdom.1252
##
## attached base packages:
## [1] grid stats graphics grDevices utils datasets methods
## [8] base
##
## other attached packages:
## [1] e1071_1.6-7 caret_6.0-68 lattice_0.20-33
## [4] irr_0.84 lpSolve_5.6.13 ggplot2_2.1.0
## [7] dplyr_0.4.3 knitr_1.12.3 tidyr_0.4.1
## [10] readr_0.2.2 checkpoint_0.3.16 RevoUtilsMath_3.2.5
##
## loaded via a namespace (and not attached):
## [1] Rcpp_0.12.4 highr_0.5.1 nloptr_1.0.4
## [4] formatR_1.3 plyr_1.8.3 class_7.3-14
## [7] iterators_1.0.8 tools_3.2.5 digest_0.6.9
## [10] lme4_1.1-12 evaluate_0.9 gtable_0.2.0
## [13] nlme_3.1-127 mgcv_1.8-12 Matrix_1.2-5
## [16] foreach_1.4.3 DBI_0.3.1 yaml_2.1.13
## [19] parallel_3.2.5 SparseM_1.7 stringr_1.0.0
## [22] MatrixModels_0.4-1 stats4_3.2.5 nnet_7.3-12
## [25] R6_2.1.2 rmarkdown_0.9.6 minqa_1.2.4
## [28] reshape2_1.4.1 car_2.1-2 magrittr_1.5
## [31] splines_3.2.5 scales_0.4.0 codetools_0.2-14
## [34] htmltools_0.3.5 MASS_7.3-45 assertthat_0.1
## [37] pbkrtest_0.4-6 colorspace_1.2-6 labeling_0.3
## [40] quantreg_5.21 stringi_1.0-1 lazyeval_0.1.10
## [43] munsell_0.4.3
