# Supplementary figures and images for: Optimized p53 immunohistochemistry is an accurate predictor of TP53 mutation in ovarian carcinoma
Source: J Pathol Clin Res. 2016 Jul 13;2(4):247–58. doi: 10.1002/cjp2.53 (PMC5091634; doi:10.1002/cjp2.53)

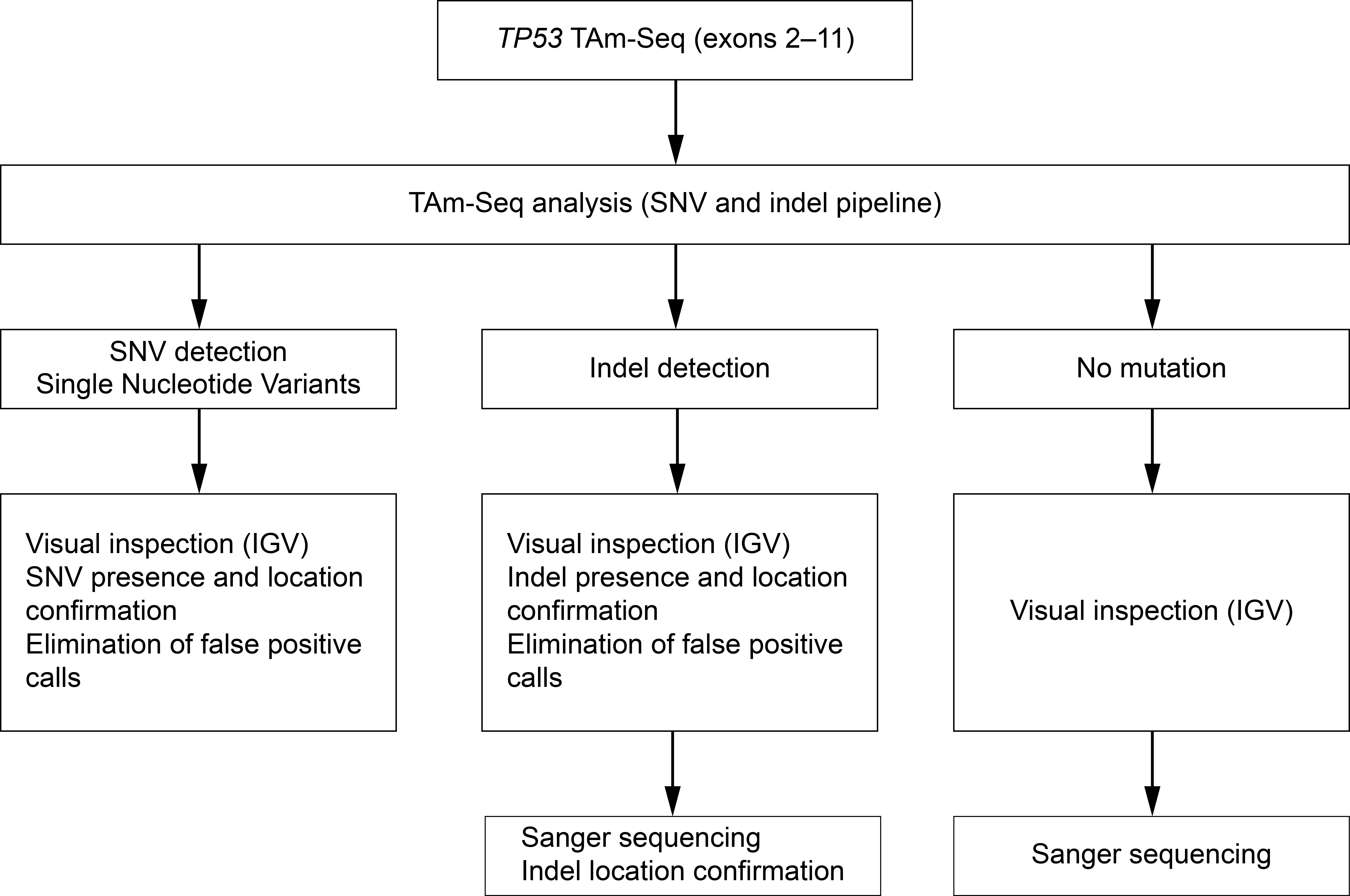

Supplement: Supplementary file 3 — Figure S1. Combined TP53 mutation detection strategy. All samples were sequenced using tagged‐amplicon sequencing. Sequencing quality control was performed by checking the coverage for each amplicon for two technical replicates and reporting samples and/or TP53 amplicons with inadequate coverage. Samples and amplicons with low or no coverage had repeat tagged‐amplicon sequencing. Visual inspection with IGV of the indexed BAM files was performed for all variants identified from the bioinformatic analysis to confirm accurate mutation calling. For sequences with ambiguous mutation start and/or end sites further Sanger sequencing was performed. If no mutation was detected by the variant calling algorithms, visual inspection of the whole TP53 coding region was performed in IGV. If a mutation was still not identifiable then Sanger sequencing was performed [file CJP2-2-247-s003.tif]

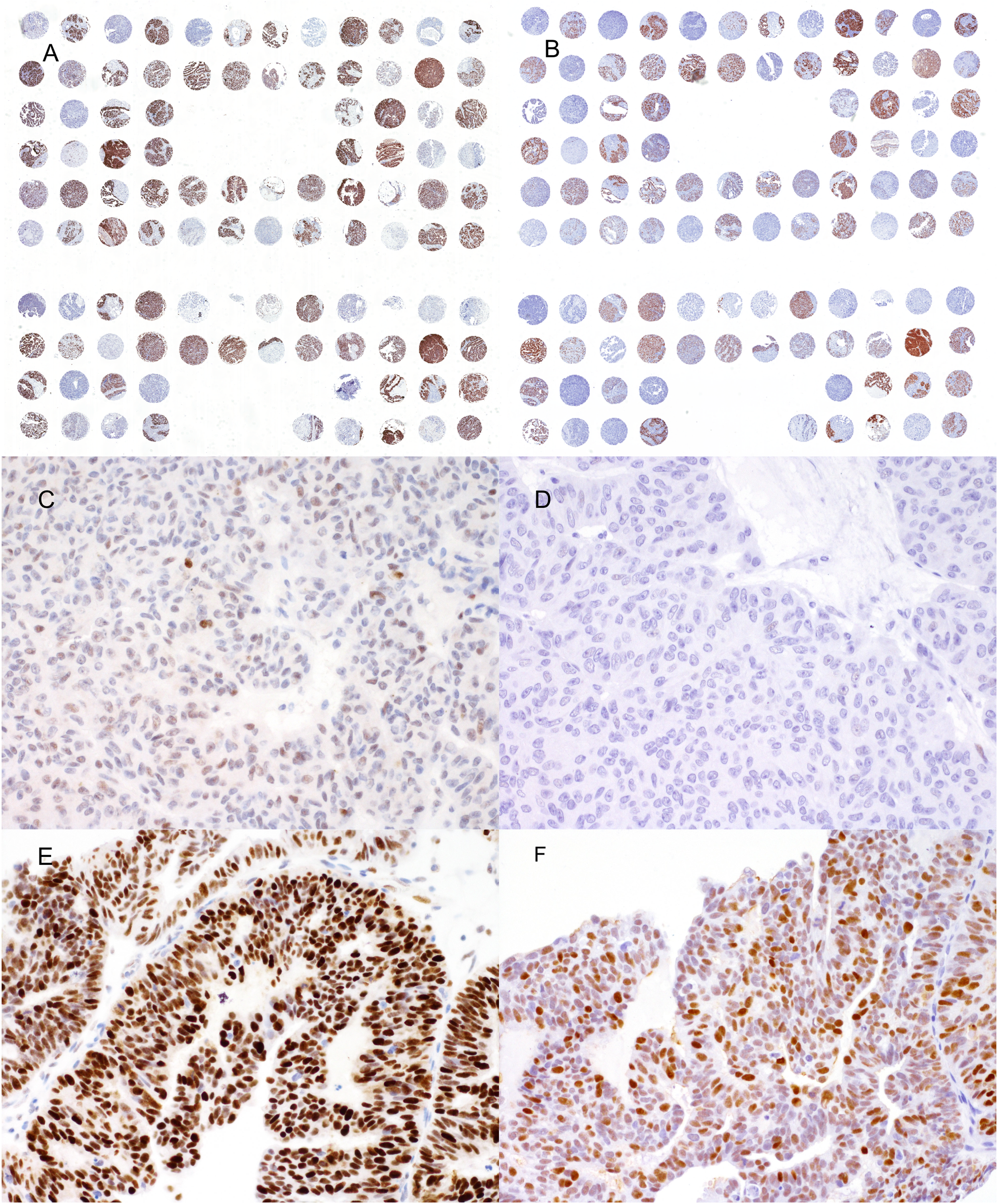

Supplement: Supplementary file 4 — Figure S2. Comparison of p53 IHC methods 1 and 4. A, B Low power view on tissue microarray shows stronger staining with method 1 (A) compared to method 4 (B) C, D EC without detectable TP53 mutation showing wild type pattern with p53 method 1 but CA with method 4 (note lack of internal control). E, F HGSOC with detected nonsynonymous mutation showing overexpression with p53 method 1 but wild type pattern with p53 method 4 [file CJP2-2-247-s004.tiff]

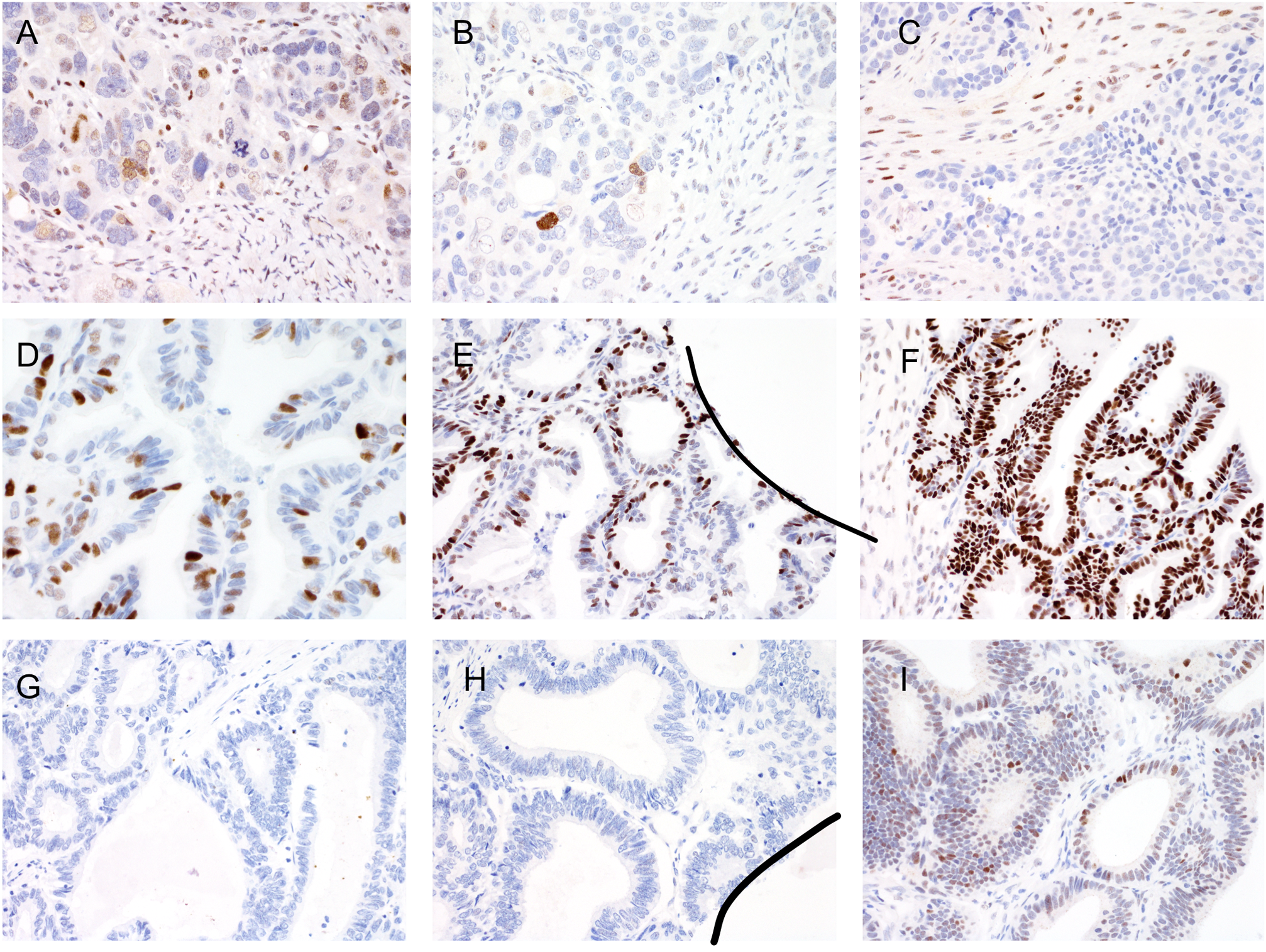

Supplement: Supplementary file 5 — Figure S3. p53 IHC quality control revisions. (A–C) HGSOC with detected splicing mutation. On tissue microarray (A) interpreted as wild type pattern that was focally present on full section (B) but predominant CA on full section, therefore revised from wild type to CA: note the presence of an internal control (C). (D–F) EC with detected nonsynonymous mutation. On tissue microarray (D) interpreted as wild type that was seen on full section adjacent to tissue microarray core hole (E, black line) but predominant overexpression on full section (F), therefore revised from wild type to overexpression. (G–I) EC without detectable TP53 mutation. On tissue microarray (G) interpreted as CA despite lack of internal control, also seen on full section adjacent to tissue microarray core hole (I, black line) but areas of wild type towards the edge of full section (I), therefore revised from CA to wild type [file CJP2-2-247-s005.tiff]

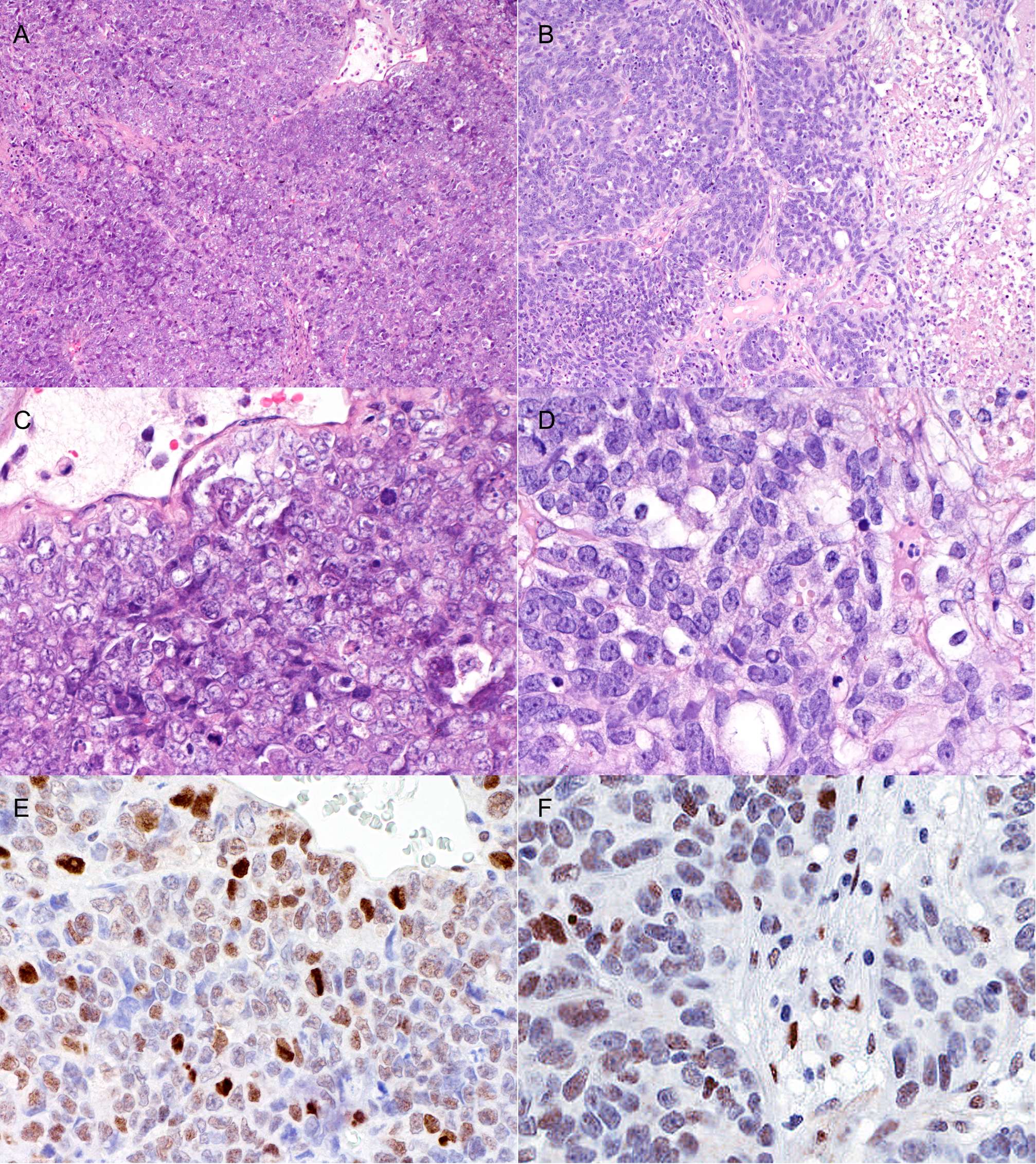

Supplement: Supplementary file 6 — Figure S4. TP53 wild type HGSOC. (A, C, E) (COEUR 22) shows a tumour with non‐specific solid architecture (A) and moderate nuclear atypia with evidence of high cell turn over (C) as well as p53 wild type pattern (E) with no evidence of TP53 mutation by sequencing. This neoplasm expressed WT1 and ARID1A (not shown) and was negative for PAX8 and ER. The patient was 47 years at diagnosis of stage IIIC disease and died 12 months after diagnosis of disease. (B, D, F) (COEUR 100) shows a tumour with solid, vaguely glandular architecture (B), spindled tumour cells with moderate nuclear atypia (D) as well as p53 wild type pattern (F) with no evidence of TP53 mutation by sequencing. This neoplasm expressed PAX8, WT1, ER and ARID1A (not shown). The patient was 85 years at diagnosis of stage IIIC disease and died 46 months after diagnosis. Both cases showed no evidence of KRAS, PTEN, PIK3CA or EGFR mutation [file CJP2-2-247-s006.tiff]

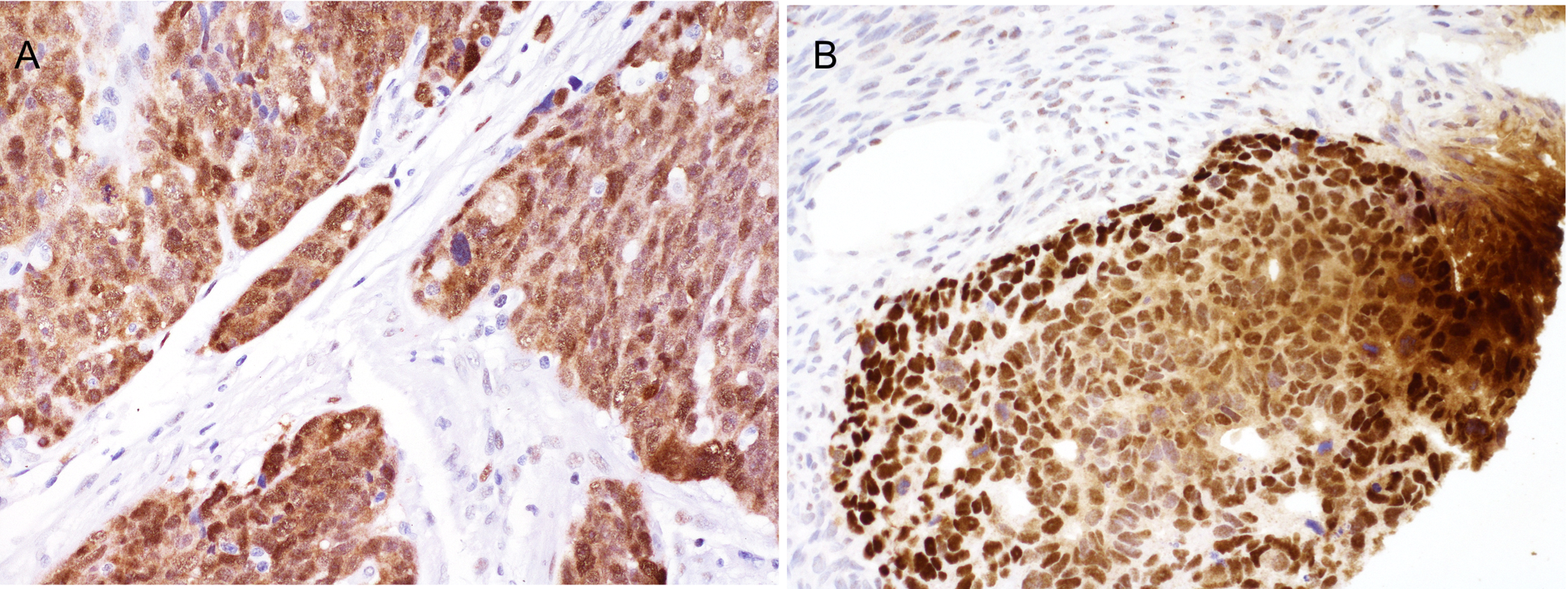

Supplement: Supplementary file 7 — Figure S5. Cytoplasmic staining. (A) HGSOC with non‐artefactual cytoplasmic staining interfering with nuclear interpretation. (B) HGSOC with artefactual cytoplasmic staining at the edge of a core due to technical artefacts [file CJP2-2-247-s007.tiff]
